# Elucidation of Ligand-Dependent Modulation of Disorder-Order Transitions in the Oncoprotein MDM2

**DOI:** 10.1371/journal.pcbi.1004282

**Published:** 2015-06-05

**Authors:** Juan A. Bueren-Calabuig, Julien Michel

**Affiliations:** EaStCHEM School of Chemistry, the University of Edinburgh, Edinburgh, United Kingdom; Indiana University, UNITED STATES

## Abstract

Numerous biomolecular interactions involve unstructured protein regions, but how to exploit such interactions to enhance the affinity of a lead molecule in the context of rational drug design remains uncertain. Here clarification was sought for cases where interactions of different ligands with the same disordered protein region yield qualitatively different results. Specifically, conformational ensembles for the disordered lid region of the N-terminal domain of the oncoprotein MDM2 in the presence of different ligands were computed by means of a novel combination of accelerated molecular dynamics, umbrella sampling, and variational free energy profile methodologies. The resulting conformational ensembles for MDM2, free and bound to p53 TAD (17-29) peptide identify lid states compatible with previous NMR measurements. Remarkably, the MDM2 lid region is shown to adopt distinct conformational states in the presence of different small-molecule ligands. Detailed analyses of small-molecule bound ensembles reveal that the ca. 25-fold affinity improvement of the piperidinone family of inhibitors for MDM2 constructs that include the full lid correlates with interactions between ligand hydrophobic groups and the C-terminal lid region that is already partially ordered in apo MDM2. By contrast, Nutlin or benzodiazepinedione inhibitors, that bind with similar affinity to full lid and lid-truncated MDM2 constructs, interact additionally through their solubilizing groups with N-terminal lid residues that are more disordered in apo MDM2.

## Author Summary

Life as we know it depends on interactions between proteins. There is substantial evidence that many interactions between proteins involve very flexible protein regions. These disordered regions may undergo disorder/order transitions upon forming an interaction with another protein. Many successful approaches to medicinal chemistry are based on mimicking the interactions of biological molecules with man-made small molecules. However how drug-like small-molecules may modulate protein disorder is currently poorly understood, largely because it is difficult to measure in details this type of interaction with experimental methods. Here we have used computer simulations to resolve with great details the process by which different small-molecules modulate the flexibility of a disordered region of the protein MDM2. This protein is overexpressed in many cancers and small-molecules that recognize MDM2 have been developed over the last decade as possible novel anti-cancer agents. We show that the flexible MDM2 “lid” region adopts different conformational states in the presence of different small-molecules. Our results suggest why some classes of small-molecules form favorable interactions with the lid region, whereas others do not. These findings may prove crucial to develop new and more effective MDM2 inhibitors, and more generally to help drug designers target disordered proteins regions with small-molecules.

## Introduction

A large fraction of proteins contain substantial regions that are unstructured in native conditions [1,2]. Protein disorder plays a key role in biomolecular function, enabling proteins to tune binding affinity and specificity to diverse partners [3]. In particular protein-complexes that involve interactions with disordered protein regions often involve disorder-to-order transitions (and vice versa) in one or both partner [4]. Nature is a rich source of inspiration in the search for new therapeutic-agents. Much successful medicinal chemistry has arisen from efforts to mimic biomolecular recognition mechanisms, prominent examples include GPCR-(ant)agonists or transition state analogue enzyme inhibitors. Likewise, there is evidence that small-molecules can productively target disordered protein regions [5]. For instance the Metallo lab has reported several small-molecule ligands that interact with disordered regions of the transcription factor c-Myc [6], though concerns about binding specificity have been raised [7]. Herbert et al. discovered an allosteric inhibitor of FGFR that induces ordering of an unstructured segment into a helical region [8]. Similar mechanisms have been inferred for allosteric inhibitors of pyruvate kinase [9]. How to anticipate productive interactions in the context of rational drug design with experimental or computational methods remains however uncertain [10], and detailed investigations are necessary to progress our understanding of this molecular recognition mechanism.

**Fig 1:**
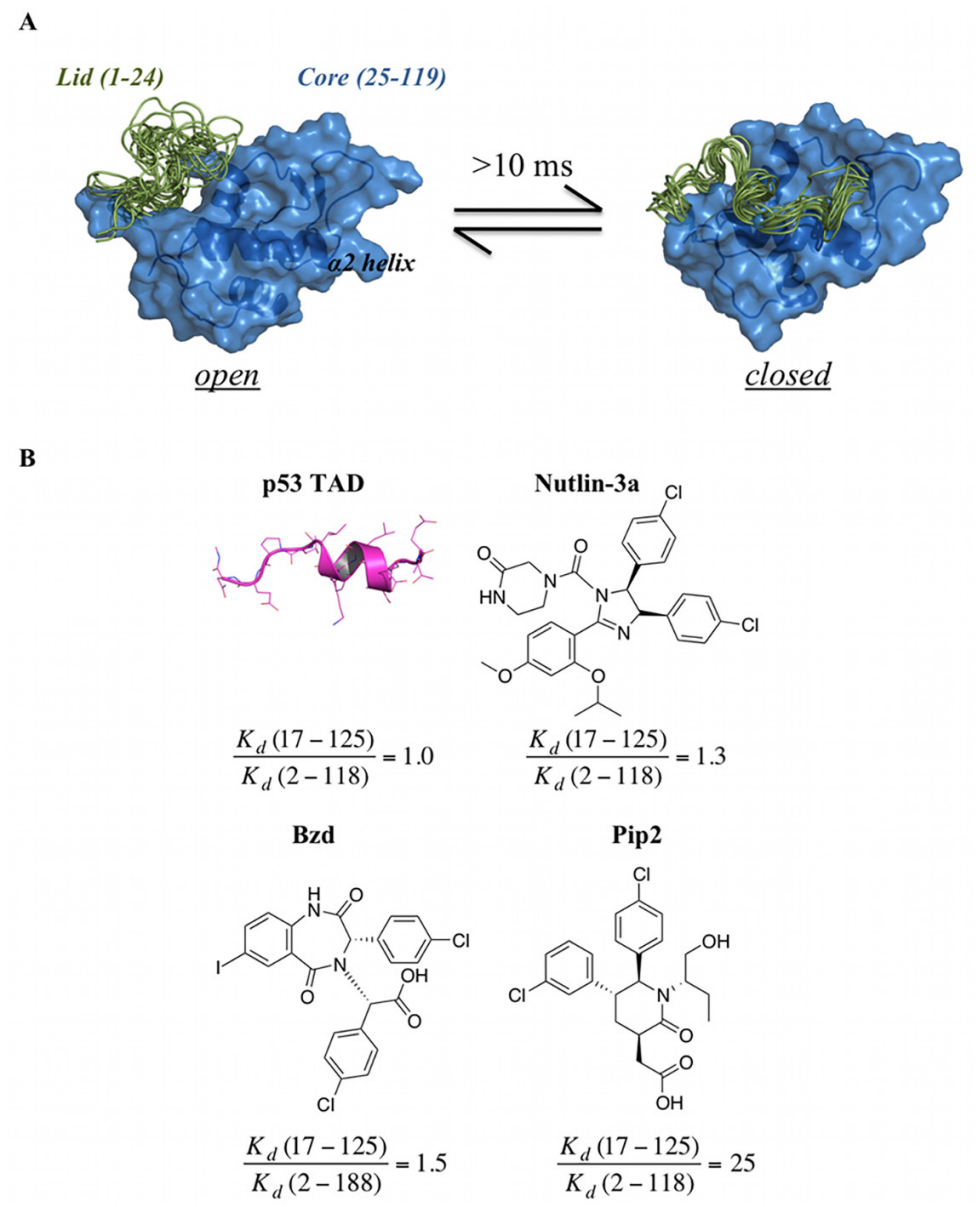
N-terminal domain of apo-MDM2 (residues 1–119) displaying several lid conformations and four representative MDM2 binders. (A) The exchange between open and closed states of the lid (1–24, in green) takes place over a 10-ms time-scale.[11–15,18] In the closed state, the lid occupies the p53-binding pocket in the MDM2 core region (25–119, in blue). The structures are representative snapshots from the umbrella sampling simulations. (B) Structure of p53 TAD (17–29) and chemical structures of three small molecule MDM2 ligands: Nutlin-3a, 1,4-benzodiazepine-2,5-dione (Bzd) and Piperidinone-2 (Pip2). Fold-improvements in binding affinity between lid containing MDM2 constructs (MDM2 (2–118) for Nutlin-3a and Pip2, MDM2 (2–188) for Bzd) and lid-less constructs (MDM2 (17–125)) are quoted below each structure and are derived from K_d_ data from Michelsen et al.[24] (isothermal titration calorimetry assay) and Parks et al.[25] (fluorescence polarization assay).

This report focusses on the consequences of small-molecule interactions with disordered protein regions, and their computational treatment. A clear illustration of the opportunities is provided by the oncoprotein MDM2. Disrupting the interaction of MDM2 with the tumor suppressor p53 is an attractive strategy in oncology [11–15]. The N-terminal domain of human MDM2 (ca. 120 residues) interacts with the transactivation domain (TAD) of p53. This interaction is mediated by Phe19, Trp23 and Leu26 of p53 that protrude into three hydrophobic pockets of MDM2 [16,17]. Additionally, the first 24 residues of the N-terminal domain of MDM2 form an unstructured flexible lid, that can adopt both “open” or “closed” states, the latter competing for the p53-binding site through a pseudo-substrate mechanism (Fig 1A) [1,2,18–20].

Ground breaking NMR studies from Showalter et al. indicated that the exchange between open and closed lid conformations occur on a >10 ms time-scale [3,18]. Potent p53/MDM2 inhibitors that bind to the p53-binding site of MDM2 have been developed, including Nutlins [4,21,22], 1,4-benzodiazepine-2,5-diones [5,23], and piperidinones [6,24] (Fig 1B). Many other classes of inhibitors have been reported, and some have progressed to clinical trials [7,25,26]. Although it has been known for some time that the lid responds differentially to large peptide-like ligands and small-molecules [18], this MDM2 region has not historically been fully considered in structure-based campaigns since the high lid flexibility hinders considerably biochemical studies and biophysical measurements [8,25]. Also, similar binding affinities to full-length MDM2 and shorter truncated lid constructs (typically 17–125) were reported for earlier inhibitors, suggesting that interactions with the lid region are of little importance to optimize binding affinity and selectivity of p53/MDM2 antagonists. However in late 2012, Michelsen et al. reported a remarkable disorder-to-order transition of the lid region upon binding of piperidinone-2 (Pip2) ligands [24], with the lid adopting a short α-helix (residues 21–24) followed by a β-turn (residues 17–20) and β-strand (residues 14–16). Significantly, unlike other ligands, the binding affinity of Pip2 towards lid-truncated MDM2 decreased by ca. 25 fold, leading Michelsen et al. to suggest that targeting this ordered MDM2 lid conformation may provide new opportunities for the design of potent and selective p53/MDM2 inhibitors.

Given the surprising outcome and high-potential for drug design purposes, clarification into the details of small-molecule lid interactions was sought for this ligand-dependent disorder-order transition. To this purpose extensive computation of atomistic lid conformational ensembles in explicit solvent for apo MDM2 and for MDM2 in complex with the four ligands depicted in Fig 1B was pursued with the aid of an enhanced molecular simulation protocol. This featured accelerated molecular dynamics (aMD) [27–29], umbrella sampling (US) [30], and variational free energy profile (vFEP) methods [31,32]. Analysis of the resulting lid structural ensembles identified significant differences in lid recognition mechanisms for the different ligands, and suggested a rationale for the high affinity of Pip2 ligands for extended-lid MDM2 constructs.

## Results

### The MDM2 lid region adopts different structural ensembles in the presence of different classes of small-molecule ligands

**Fig 2:**
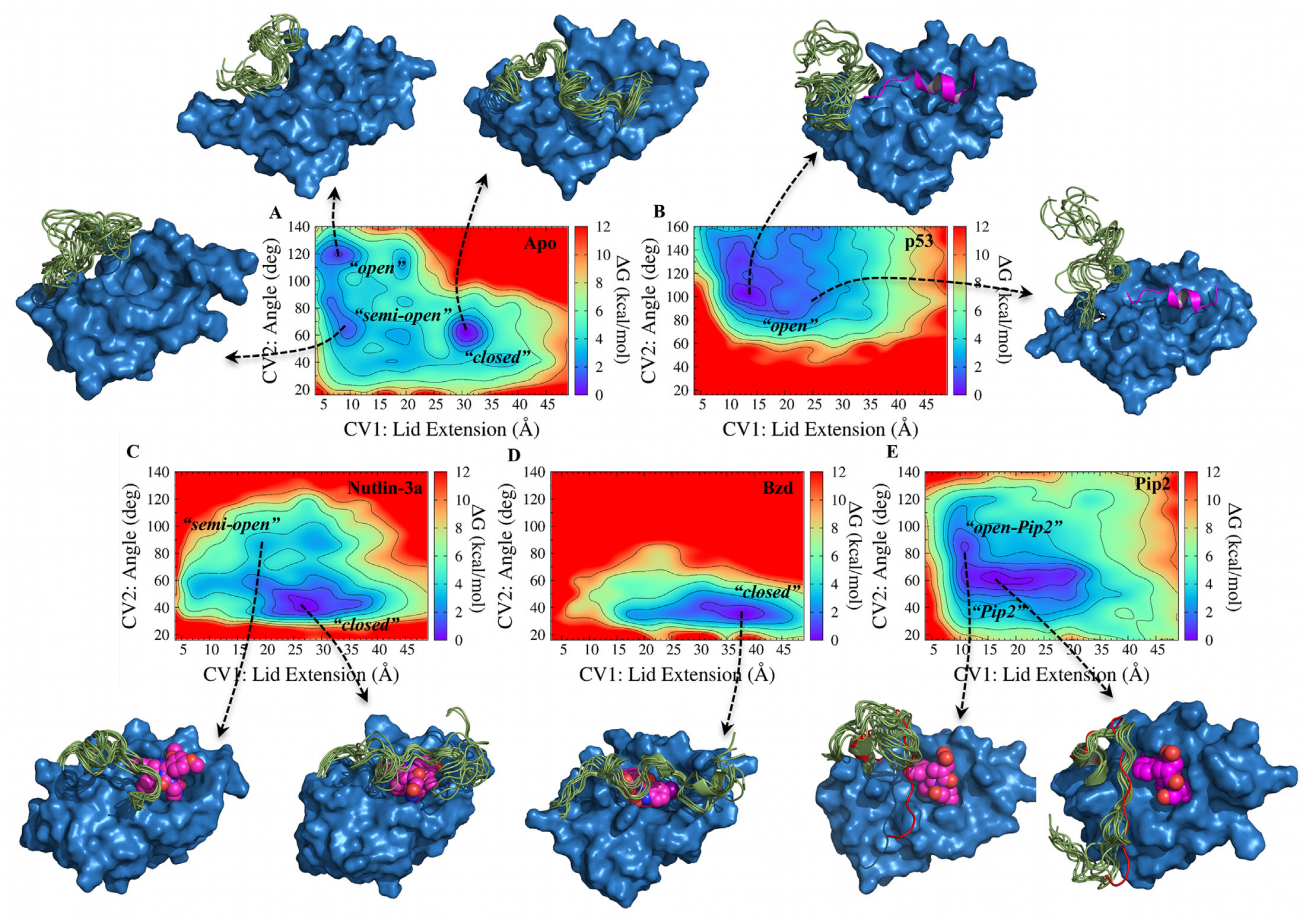
Ligand-dependent modulation of MDM2 lid free energy landscapes. CV1 (lid extension) in Å, and CV2 (lid-core angle) in degrees. Free energy contours (kcal mol^-1^) are shown as a color coded heat map. Free energies are relative to the lowest free energy bin and are shown up to 12 kcal mol^-1^ above the lowest free energy bin. For every system, representative structures of MDM2 displaying 10 lid conformations from selected bins are shown. A) apo MDM2. B) p53-TAD(17–29)/MDM2 C) Nutlin-3a/MDM2 D) Bzd/MDM2 E) Pip2/MDM2. The lid conformation seen in the x-ray structure of MDM2 (6–125) reported by Michelsen et al. is shown in red cartoon representation [24].

As expected in light of the anticipated time scale for transitions between open and closed lid states, unbiased equilibrium MD simulations on the timescale of several hundreds of ns of apo MDM2, initiated from a range of different initial lid conformations, failed to generate a single transition from open to closed states of the lid (S1 Fig). By contrast the aMD simulation protocol sampled transitions between open and closed apo lid states in simulations of ca. 100 ns (S1 Fig). In the simulations of small molecule bound complexes, complete lid opening was not observed with cMD or aMD protocols, but enhanced conformational fluctuations were observed with the latter protocol (S1 Fig). Though some variability is apparent and no low-dimensionality projection is fully satisfactory for such complex system, the present protocol enabled the detection of significant differences between the different complexes, and a broad range of lid conformations were observed (Fig 2).

**Fig 3:**
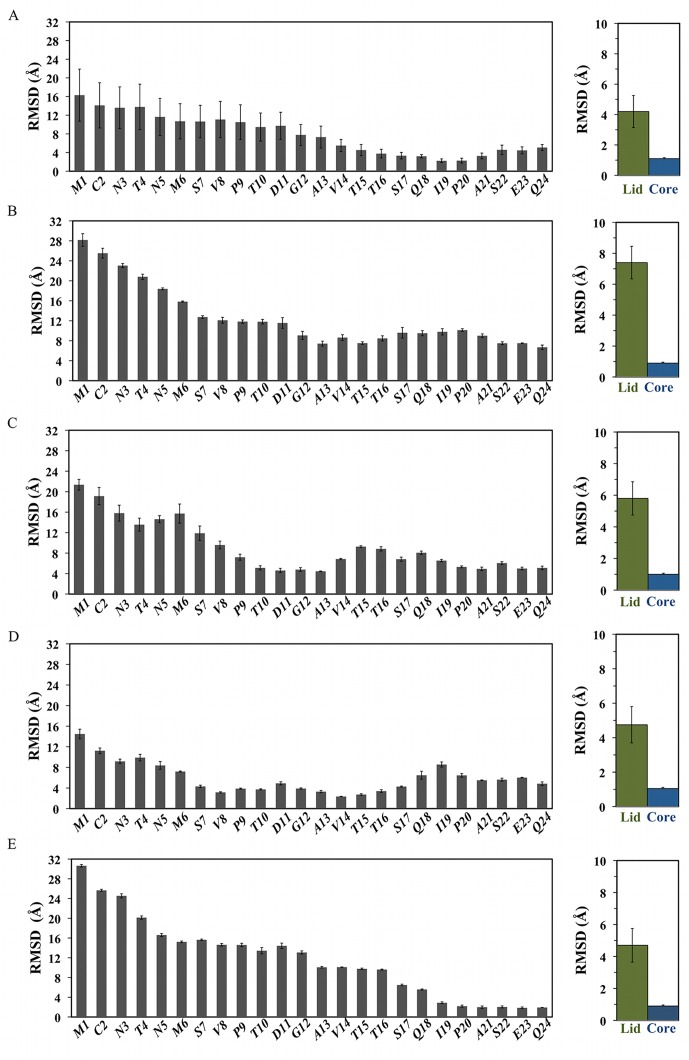
Ligand-dependent modulation of MDM2 lid flexibility. Left) per-lid residue average backbone RMSD. Right) Backbone RMSD over full lid (green) or core (blue) regions. From top to bottom, A) apo MDM2, B) p53-bound MDM2, C) Nutlin-3a-bound MDM2, D) Bzd-bound MDM2, E) Pip2-bound MDM2.

**Fig 4:**
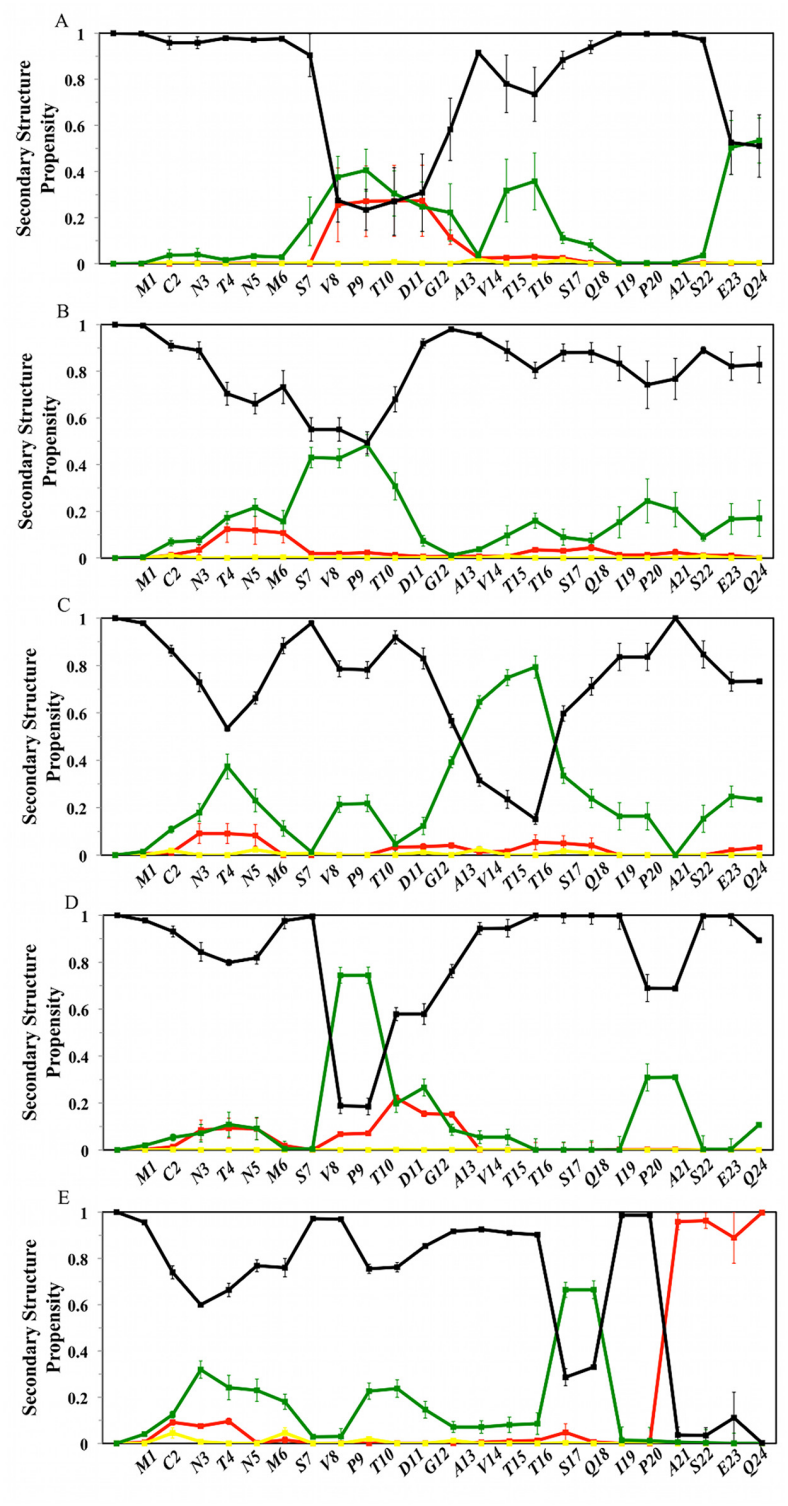
Ligand-dependent modulation of MDM2 lid secondary structure propensity. Red: helix; Green: Turn; Yellow: β-Strand. Black: Coil. Secondary structure definitions follow the DSSP code [34]. From top to bottom, A) apo MDM2, B) p53-bound MDM2, C) Nutlin-3a-bound MDM2, D) Bzd-bound MDM2, E) Pip2-bound MDM2.

**Fig 5:**
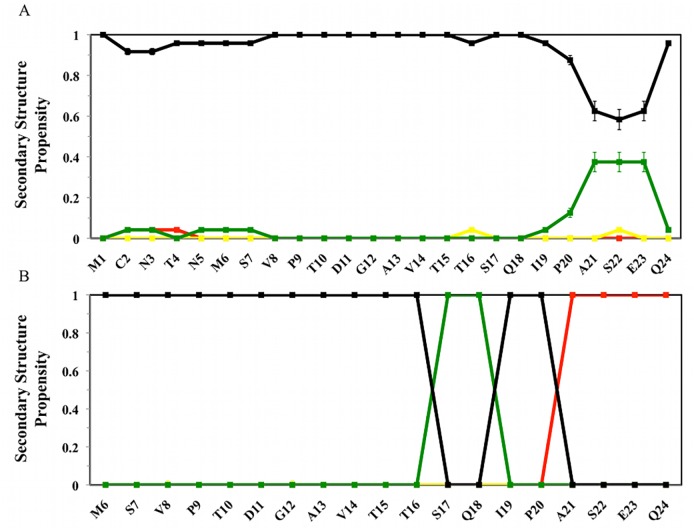
Secondary structure propensity of the lid region from experimental methods. A) NMR ensemble of apo MDM2 lid (1–24) from Uhrinova et al [19]. B) crystal structure of Pip2-MDM2 lid (6–24) from Michelsen et al.[24]. Red: helix; Green: Turn; Yellow: β-Strand. Black: Coil.

Uncertainties in the computed free energy surfaces for each system were assessed by monitoring convergence over regular time-intervals (S2–S3 Figs). Equilibrium properties of the ligand-bound complexes are reasonably reproducible, with greater uncertainties observed for the most flexible lid residues in apo MDM2 (Figs 3–5). Three major low free energy regions were identified in the FES of apo MDM2 (Fig 2A). The lowest corresponds to a “closed” state (CV1 = 31 Å; CV2 = 62°), with the lid adopting a semi-extended conformation in contact with α2 helix residues. While this conformation would hinder binding of the p53 TAD as a result of steric clashes with the lid, the Phe19-Trp23-Leu26 cleft was still accessible to small molecules such as Nutlins. The second region (CV1 = 7 Å; CV2 = 119°) corresponds to an “open”, compact state of the lid. In this state, the p53-binding site is fully accessible to large and small ligands. No open, extended lid conformations were observed, thus the lid in the open state adopts collapsed structures. A third additional region (CV1 = 10 Å; CV2 = 64°) corresponds to an intermediate “semi-open” state. In this conformation, the lid approaches the core of MDM2. Although the hydrophobic pocket was still fully accessible to small ligands, binding of the larger p53 TAD would be hindered.

On the basis of NMR spin relaxation measurements, Showalter et al. found that apo MDM2 favors a closed state over an open state by ca. 1.2 kcal/mol. While the definition of an open or closed state is somewhat arbitrary, comparison with the present results was pursued by classifying each bin in the computed two-dimensional free energy surface as “open” or “closed”. Defining a closed state for bins with a cutoff >23 Å for the lid extension and <80° for the lid-core angle collective variables, the closed state is favored over the open state by 0.3±0.1 kcal/mol. Varying this cutoff by ±3 Å and ±6° has little effect on the result (±0.1 kcal/mol). Thus the simulations predict a higher population of open states than the NMR data, though the agreement is still reasonable. This finding is consistent with reports from Best and co-workers that have found unfolded conformational ensembles to be too compact and stabilized by residual secondary structures with current classical force fields [33,34]. The p53 TAD-MDM2 simulations (Fig 2B) revealed a single broad free energy basin corresponding to a range of fully “open” and compact conformations (CV1 = 12 Å; CV2 = 105°). Due to the presence of the p53 peptide in the hydrophobic pocket, the lid is displaced towards a broad range of open disordered states. Nevertheless, the most stable lid conformations detected for the p53 TAD-MDM2 complex were similar to the most open conformations shown in the apo-MDM2 system, both displaying a lid core angle of ca. 120° and a compact state of the lid (CV1 ca. 10 Å).

A low free energy basin (Fig 2C) corresponding to a “closed”, extended state was found for Nutlin-3a/MDM2 (CV1 = 27 Å; CV2 = 41°). In comparison with apo MDM2, the occurrence of open lid state is not only decreased, but the closed state differs in nature, adopting even lower lid-core angle values. This is because the position of the lid in the closed state shifts from contacting helix α2 to cover Nutlin-3a. Thus, Nutlin-3a does not compete with the lid for access to the p53-binding site, and stabilizes a different “closed” lid state than observed in apo MDM2. An additional local minimum, corresponding to “semi-open” conformations, was also observed for the Nutlin-3a/MDM2 complex, with the lid region still interacting with the ligand but in a more compact conformation. In the presence of Bzd, a change in favored “closed” lid state is also observed (Fig 2D). In this case the FES presented a single free energy basin (minimum at CV1 = 38 Å; CV2 = 37°) that corresponds to a “closed” and fully extended lid conformation over the hydrophobic cleft. The lid extension in this case was ca. 10 Å greater than that observed in low free energy conformations in the apo and Nutlin3a-MDM2 systems. The lid may readily adopt even lower CV2 angle (<40°) values than with Nutlin-3a, because the smaller size of the Bzd ligand enables the lid to close further the p53 binding cleft. The FES of MDM2 in complex with Pip2 displayed a major free energy basin (CV1 = 16 Å; CV2 = 62°) indicative of a relatively compact “Pip2” lid state (Fig 2E) that significantly differs from the previously observed states. Lid conformations in this region of the FES contain a short ordered α-helix at residues 21–24, a sharp bend around residues 17–20, and residues 10–16 adopt an extended conformation. This lid conformation is consistent with the X-ray and NMR data reported by Michelsen et al. for MDM2 (6–125) [24]. A secondary minimum is also apparent (CV1 = 10 Å; CV2 = 85°), in this “open-Pip2” conformation the lid still contains a short α-helix at residues 21–24, but segment 1–16 adopts a collapsed rather than extended conformation. Taken together, these results indicate that the conformational preferences of the MDM2 lid region are remarkably influenced by the chemical structure of the different classes of small-molecule MDM2 antagonists, and that multiple distinct closed states are readily achieved by the MDM2 lid region.

### Impact of ligand binding on lid dynamics

#### Ligand binding maintains or even increases overall lid flexibility

Quantitative insights into lid interactions behavior was pursued by computing flexibility profiles for every lid residue. Lid-backbone RMSD calculations indicate a trend for decreased flexibility along the lid sequence, and this is apparent for all systems (Fig 3).

Similar trends were observed when all heavy-atom RMSD values were computed (S4 Fig). As expected, overall the lid is considerably more flexible than the core region. Comparison with the apo results (Fig 3A) indicates that p53 binding generally increases lid flexibility, in agreement with the notion that suppression of the closed lid states increases lid disorder (Fig 3B).[18] Nutlin-3a (Fig 3C) and Bzd (Fig 3D) decrease the lid flexibility in intermediate segments that were substantially disordered in apo (residues 9–14 and 7–17 respectively). Intriguingly, the lid in Nutlin-3a bound MDM2 is nevertheless on average slightly more disordered than in apo. Thus lid residues in close contact with the ligand exhibit decreased flexibility, whereas the remainder of the lid shows increased flexibility. On the other hand, similar overall average lid flexibility (within error bars) is seen for Bzd-bound MDM2. By contrast, Pip2 (Fig 3E) significantly orders residues at the base of the lid (residues 20–24). These residues were already among the most ordered in apo MDM2. However Pip2 also significantly increases the flexibility of the lid residues at the beginning of the sequence, and overall the lid flexibility is comparable to apo MDM2. Taken together these results demonstrate that, although the binding of small-molecules considerably reduces the flexibility of some segments of the lid region, the flexibility of the lid region is overall maintained or even increased. ITC measurements reported by Michelsen et al. suggest that Pip2 ligands have more unfavorable binding entropy for MDM2 constructs that include the lid, versus those that lack this region [24]. By contrast Nutlin ligands have similar binding entropy for lid and lidless MDM2 constructs. This was interpreted by Michelsen et al. as evidence that MDM2 lid ordering by Pip2 is entropically unfavorable. The present results complicate this analysis since the base of the lid region is shown to have decreased flexibility in presence of Pip2, but this is overall offset by an increase in lid flexibility at the N-terminal region of the lid. The issue is not fully addressed here since converging precise and accurate conformational entropy estimates from MD simulations remain challenging for large disordered protein regions, and RMSDs provide a convenient albeit approximate metric to estimate conformational entropy changes. Additionally it isn’t possible to de-convolute from the ITC data changes in protein entropy from changes in solvent entropy, which could also account for a large fraction of the overall entropy changes upon binding.

### Impact of ligand binding on lid structure

#### Ligand binding induces turn or helical segments in the lid region

Secondary structure propensities for every lid residue are depicted in Fig 4 [35,36].

In the apo structure, the lid displays small α-helical propensities near residues 9–13, noticeable turn propensities near residues 8–13, 15–16 and 23–24, with other regions predominantly in a coil state (Fig 4A). In the NMR data of apo MDM2 from Uhrinova et al.,[19] a noticeable turn propensity was only observed at the base of the lid (Fig 5A).

There is less evidence for strong secondary structure preferences in the lid region when the p53 peptide is bound to MDM2, although a small propensity for turns between residues 8–11 is maintained (Fig 4B). Interestingly, in Nutlin-3a-bound MDM2, the lid shows a strong tendency to form a turn region between residues 13–17 (Fig 4C), whereas a short turn region is frequently seen near residues 9–10 for Bzd-bound MDM2 (Fig 4D). Thus the lid responds to different ligands by increasing turn propensities at specific lid positions. In the Pip2-MDM2 complex, a α-helix (21–24) and turn (17–18) motif is clearly identified (Fig 4E), thus explaining the significant decrease in flexibility seen at the base of the lid in Fig 3E. This motif is also apparent in the crystallographic data of Michelsen et al [24] (Fig 5B). By contrast, the β-strand motif around residues 14–16 that was discussed by Michelsen et al. is not observed in the simulations. Neither is this motif detected by the DSSP algorithm when applied to the crystallographic structure reported by Michelsen et al. (Fig 5B), Thus even though the Pip2-bound lid backbone conformations of residues 14–16 present in the low free energy regions of the computed FES (Fig 2E) broadly match the lid conformations seen in the experimental data, there is some ambiguity in the assignment of a given secondary structure to this lid segment.

### Small-molecules engage the lid with different patterns of hydrophobic and polar interactions

Further insights into these intriguing results are gained by qualitative inspection of equilibrium lid intermolecular and intramolecular interaction patterns. Attention was focused on lid residues that exhibit an average number of hydrophobic contacts or hydrogen bonds that is significantly above typically observed values. Cutoff values of 4 hydrophobic contacts or 0.25 hydrogen bonds for intermolecular interactions, and 4 hydrophobic contacts or 1.0 hydrogen bond for intramolecular lid interactions were deemed sufficient to identify the most significant interactions. In addition, the observed contacts were mapped to specific MDM2 core residues and ligand functional groups involved in interactions with the lid. This was done by generating and visualizing representative structural ensembles of each complex by resampling of computed conformations according to their equilibrium probabilities.

#### Apo MDM2

**Fig 6:**
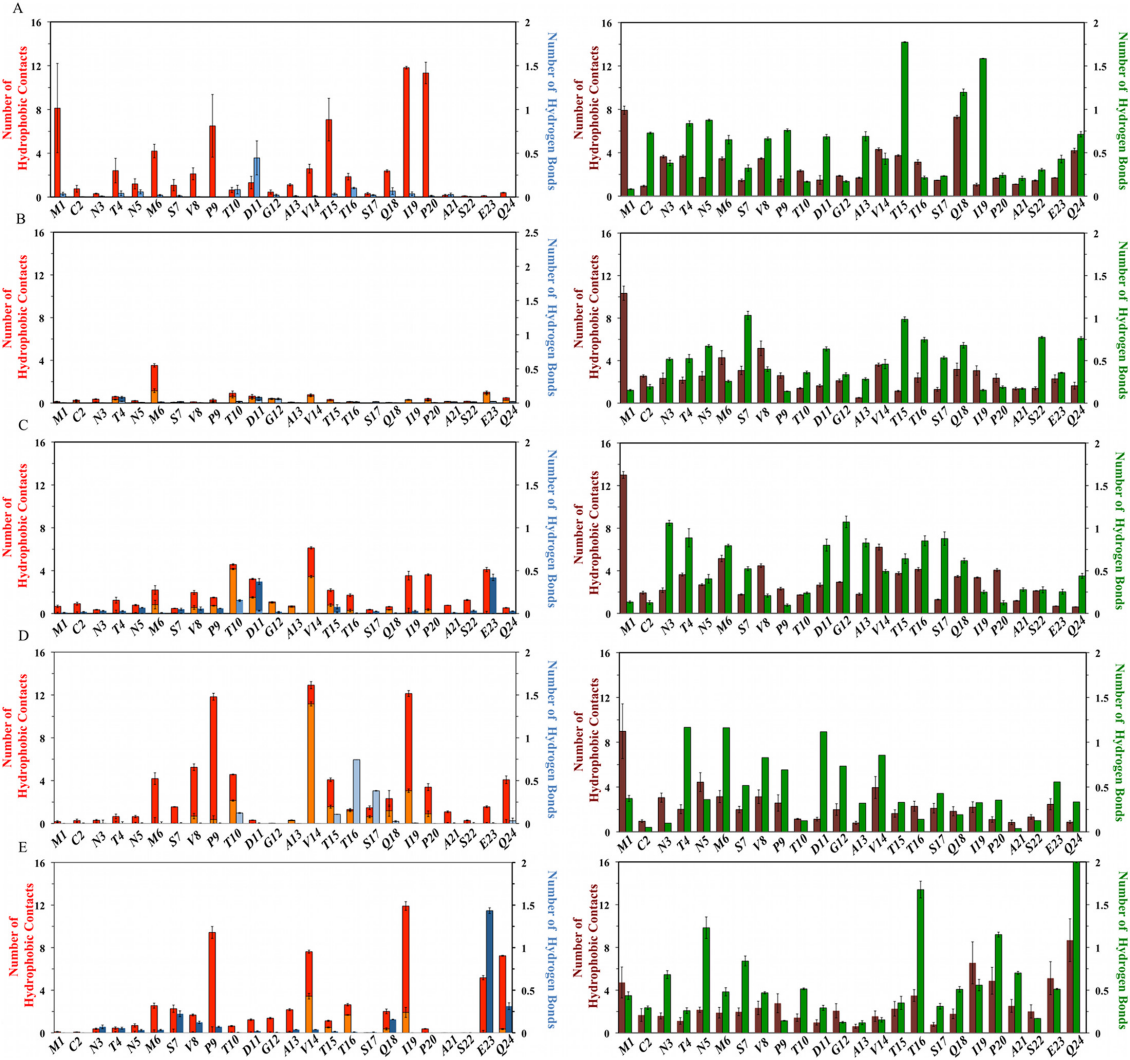
Ligand-dependent modulation of MDM2 lid intermolecular and intramolecular interactions. Lid-core hydrophobic contacts are displayed in red; lid-ligand hydrophobic contacts are displayed in orange; lid-lid hydrophobic contacts are displayed in maroon. Lid-core hydrogen bonds are displayed in dark blue; lid-ligand hydrogen bonds are displayed in light blue; lid-lid hydrogen bonds are displayed in green. From top to bottom, A) apo MDM2, B) p53-bound MDM2, C) Nutlin-3a-bound MDM2, D) Bzd-bound MDM2, E) Pip2-bound MDM2.

Under these conditions, apo MDM2 (Fig 6A, left panel) shows significant lid-core contacts involving Met1, Met6, Pro9, Thr15, Ile19, Pro20 and lid-core hydrogen bond involving Asp11.

**Fig 7:**
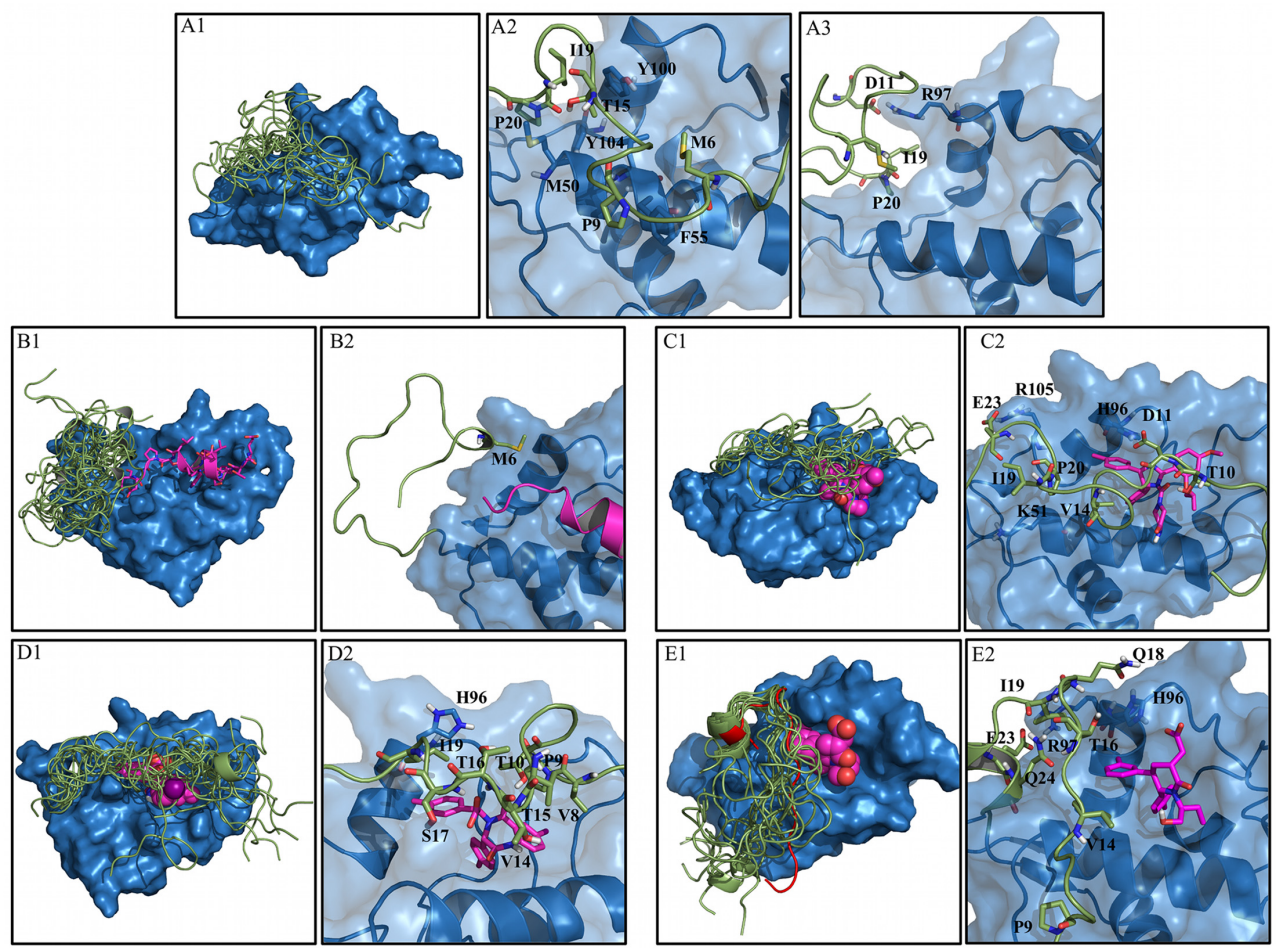
MDM2 lid structural ensembles and contact details from representative structures for the five systems studied. A) Apo-MDM2. Structural ensemble (A1) and structural details of the closed (A2) and open states (A3). B) p53-MDM2 complex. Structural ensemble (B1) and structural details (B2). C) Nutlin3a-MDM2 complex. Structural ensemble (C1) and structural details (C2). D) Bzd-MDM2 complex. Structural ensemble (D1) and structural details (D2). E). Pip2-MDM2 complex. Structural ensemble (E1) and structural details (E2). The X-ray structure of Pip2-MDM2 from Michelsen et al. is shown in red cartoon.[24] Representatives PDB files are included in the Supporting Information.

The lid-core hydrophobic contacts arise mainly from the closed state conformations, whereas the hydrogen bond involving Asp11 is seen in the open state conformations. The significant lid-lid hydrophobic contacts from Met1, Val14 and Gln18 originate from open and compact lid conformations, whereas the significant hydrogen-bonding interactions for lid residues Thr15 and Ile19 arise mainly from closed conformations (Fig 6A, right panel). Fig 7A1. depicts a representation of the apo MDM2 structural ensemble that highlights the greater disorder of open versus closed lid states.

Fig 7A2 shows that the majority of the lid contacts in the closed state involve helix α2, whereas Asp11 engages Arg97 to stabilize the open state (Fig 7A3). Ile19 and Pro20 adopt the same conformation in both open and closed states, interacting with Tyr100 and Tyr104, thus explaining their relative low flexibility seen in Fig 3A.

#### p53-bound MDM2

With the exception of Met6, the p53-bound MDM2 lid (Fig 6B, left panel) shows no significant lid-core or lid-ligand contacts. This is not balanced by a significant increase in lid-lid interactions in comparison with apo MDM2 (Fig 6B, right panel), explaining why the lid region is significantly more disordered when MDM2 is bound to p53. Consequently in the p53 TAD bound complex the lid structural ensemble is largely shifted to an open state (Fig 7B1). The only significant contacts involve Met6 with multiple residues from the core (Fig 7B2).

#### Nutlin-3a bound MDM2

When bound to Nutlin-3a, (Fig 6C, left panel) the lid exhibits hydrophobic contacts with Thr10 (mainly ligand contacts), Val14 (ligand contacts), Ile19, Pro20, Glu23. Hydrogen-bonding interactions are observed with Glu23 and Asp11 (with the core) and to a lesser extent Thr10 (with the ligand). Bulky hydrophobic lid residues in the N-terminal region of the lid (Met1, Met6, Val8, Val14) are frequently in contact with other lid residues (Fig 6C, right panel). The structural ensemble for the Nutlin-3a complex (Fig 7C1) captures the trend for lid ordering around residues 10–15. Hydrogen bonding interactions (Fig 7C2) seen in Fig 6C predominantly involve Glu23 with Arg105, Asp11 with His96 and Thr10 with the Nutlin-3a imidazoline carbonyl group. Val14 is in contact with Leu54 and the chloro-phenyl ring of Nutlin-3a, while Ile19 and Pro20 approach helix α2 and are in contact with Lys51.

#### Bzd bound MDM2

When bound to Bzd (Fig 6D, left panel) the lid shows substantial contacts with Met6, Val8, Pro9, Thr10, Val14 (mainly with the ligand), Thr15, Ile19, Gln24. Significant hydrogen-bonding interactions are observed with Thr16 and Ser17 (with the ligand). Hydrogen-bonding interactions between lid residues are more frequently observed in the N-terminal region of the lid, notably Thr4, Met6, Asp11 (Fig 6D, right panel). In agreement with Fig 3D, the Bzd-bound lid conformational ensemble (Fig 7D1) indicates significant decrease in lid flexibility around residues 8–18. This correlates with the several hydrophobic contacts recorded in Fig 6D. Although Asp11, Gly12 and Ala13 were significantly ordered, these lid residues remained solvent-exposed and no significant additional interactions with core residues or ligand functional groups were observed. The backbone NH of Thr16 and Ser17 interact with the carboxylate group of Bdz (Fig 7D2), whereas less frequent hydrogen bonding interactions of Thr10 and Thr15 involved the Bzd ring. Val14 is frequently in contact with the benzodiazepine ring. Pro9 and Val8 interact with Met62 from the α2 helix while Ile19 is both in contact with the chloro-phenyl ring of Bzd and with the His96 imidazole ring.

#### Pip2 bound MDM2

**Fig 8:**
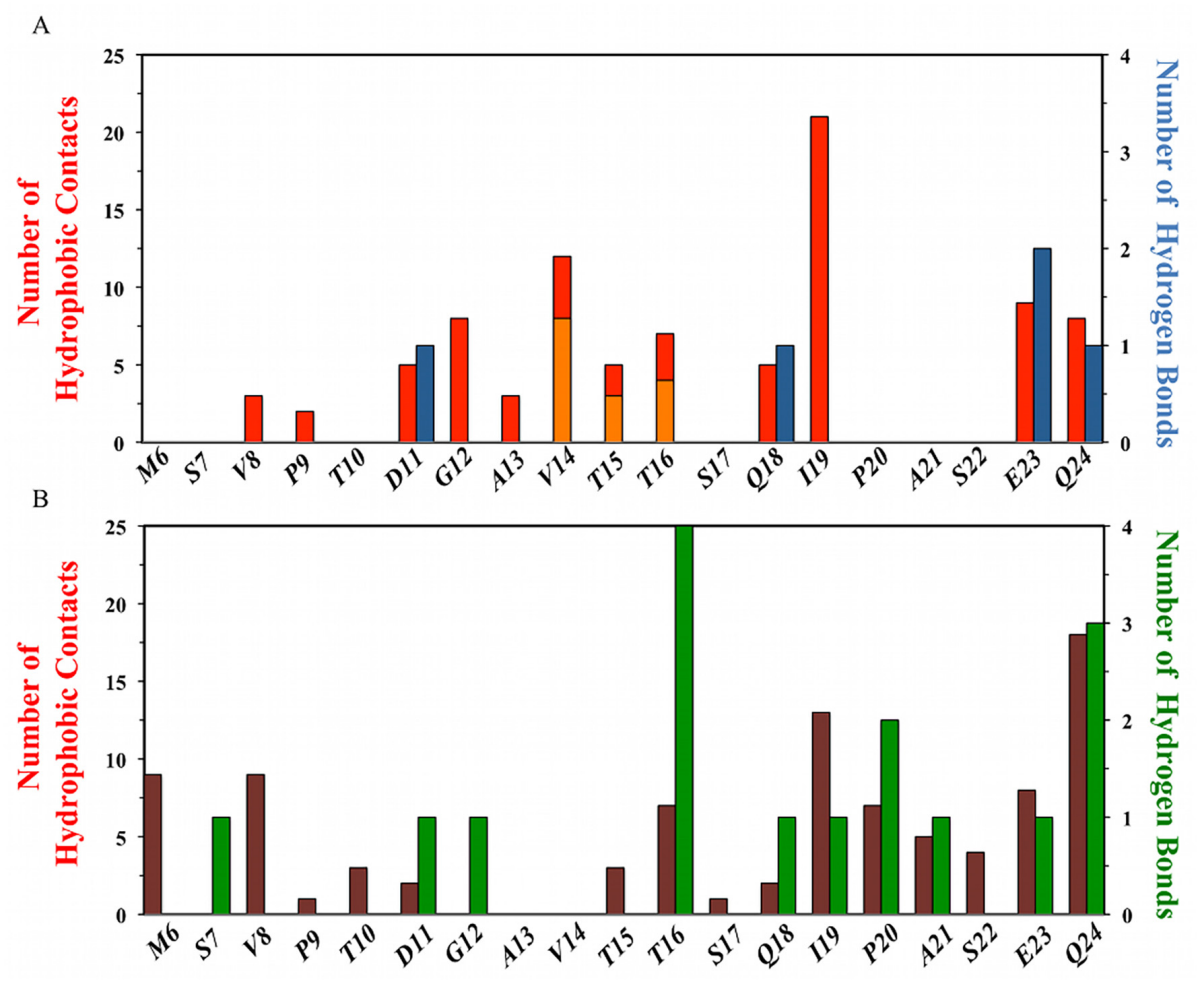
MDM2 lid intermolecular and intramolecular interactions observed in the Pip2-MDM2 crystal structure of Michelsen et al [24]. Top) Lid-core hydrophobic contacts are displayed in red and lid-ligand hydrophobic contacts, in orange. Lid-core hydrogen bonds are shown in dark blue and lid-ligand hydrogen bonds in light blue. Bottom) Lid-lid hydrophobic contacts are displayed in maroon. Lid-lid hydrogen bonds are displayed in green.

Finally, when bound to Pip2 (Fig 6E, left panel), the lid shows strong contacts with Pro9, Val14, Ile19, Glu23, Gln24. Significant hydrogen-bonding with core residues interactions involve Glu23 and Gln24. In comparison with Nutlin-3a and Bzd, the major qualitative difference is that the majority of the contacts occur towards the C-terminal region of the lid. Inspection of the intramolecular interaction patterns (Fig 6E, right panel) also reveals that the majority of the significant lid-lid contacts (Ile19, Pro20, Glu23, Gln24) and hydrogen-bonding lid-lid interactions (Thr16, Pro20, Gln24) also occur near the C-terminal region of the lid. This interaction pattern matches well with the interaction pattern observed in region 13–24 of the X-ray structure of Michelsen et al.[24], although the number of contacts or hydrogen-bonding interactions per residue are usually greater in the crystallographic structure (Fig 8).

This likely reflects the different environments experience by the lid, i.e. the lid is flexible in the simulations that reproduce solution conditions, but is ordered in the X-ray structure because of crystal packing contacts and of the temperature at which the X-ray diffraction patterns were collected. The Pip2 bound structural ensemble (Fig 7E1) recapitulates well the trends seen in Fig 3E, with a significant ordering around residues 19–24 that adopt an α-helical conformation, and increased flexibility in N-terminal regions of the lid. Fig 7E2 indicates that the lid α-helix is stabilized by a salt-bridge between Glu23 and Arg97. Strikingly, unlike Nutlin-3a and Bzd solubilizing groups, the carboxylate group of Pip2 was found to exclusively form hydrogen-bonding interactions with the MDM2 core residue His96. This interaction appears somewhat stabilized by occasional hydrogen-bonding interactions between Nδ of His96 and Oε of Gln18. The *meta*-chloro phenyl group of Pip2 also forms contacts with Val14 and Thr16, as observed in the X-ray structure of Michelsen et al [24], Pro9 makes contact with Glu52 and Phe55 from the α2-helix, while Ile19 and Gln24 interact with Tyr100.

Overall the results indicate that the pattern of lid-core interactions observed in apo MDM2 are strongly perturbed by the binding of all ligands, and that each of the four ligands studied here form a distinct pattern of interactions with the lid.

### Impact of ligand binding on lid energetics

#### Pip2 stabilizes lid-core interaction energetics

**Fig 9:**
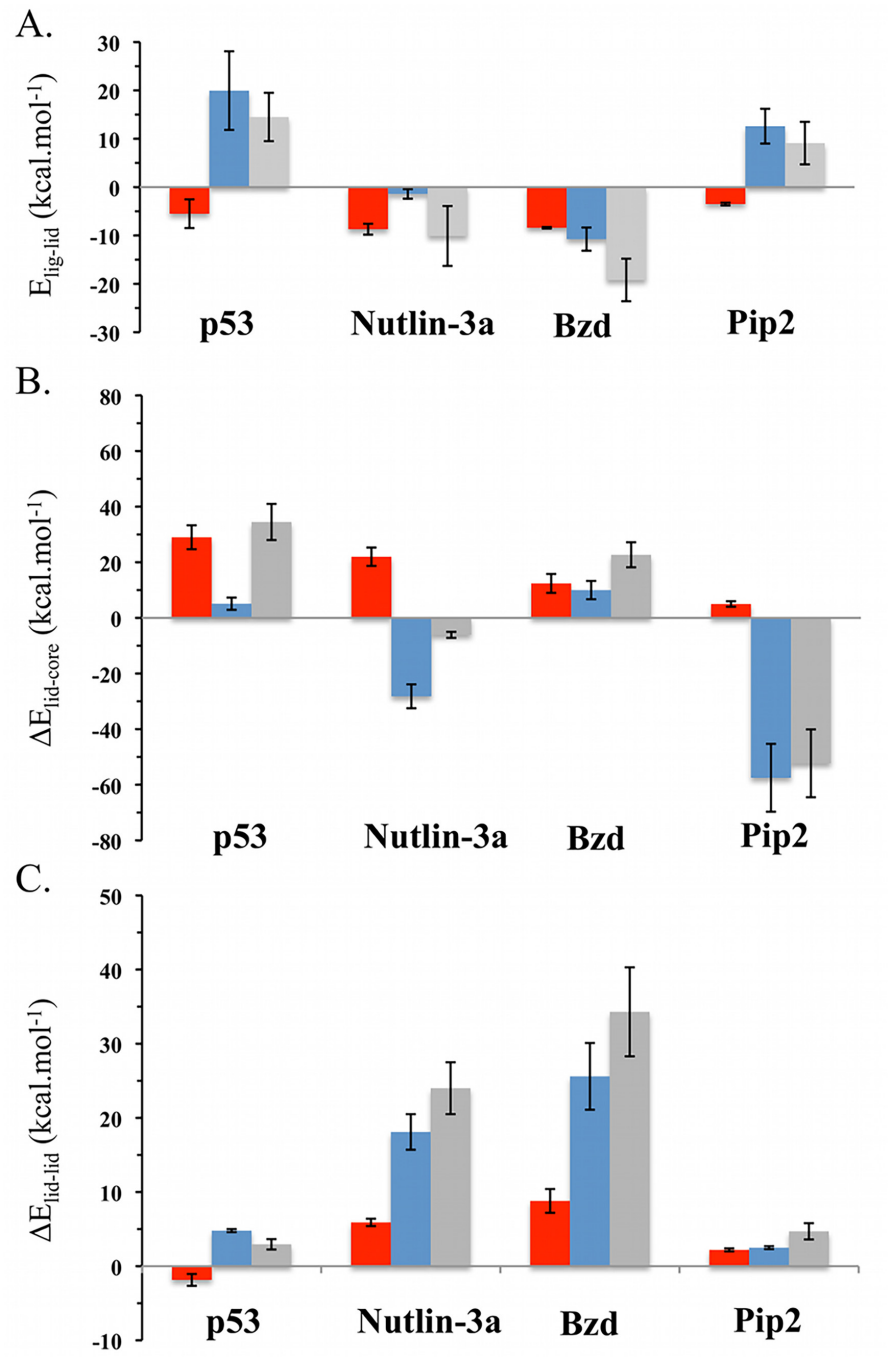
Impact of ligand binding on MDM2 lid energetics. A) Ensemble average of lid-ligand interaction energies *E*_*lig-lid*_ = <*E*_*lig-lid*_>. B) Changes in ensemble average of lid-core interaction energies Δ*E*_*lid-core*_ = <*E*_*lid-core*,*holo*_>—<*E*_*lid-core*,*apo*_>. C) Changes in ensemble average of lid-lid interaction energies Δ*E*_*lid-lid*_ = <*E*_*lid-lid*,*holo*_>—<*E*_*lid-lid*,*apo*_>. Lennard-Jones energies are depicted in red, Coulombic energies in blue, and total interaction energies in gray. Energies are shown in kcal.mol^-1^.

Further insights into the improved binding affinity of Pip2 for lid-including constructs were sought by evaluation of lid energetics. Full consideration of binding energetics was not pursued here as solvation free energy estimates from the reweighted trajectories were found to be too imprecise to enable meaningful comparison between ligands. Further as discussed before, changes in conformational entropies of the lid region were not computed. Thus attention was focused on lid interaction energy components as these were found to be sufficiently well converged to enable meaningful comparisons of energetic profiles between different ligands. Fig 9A depicts lid-ligand interaction energies.

As expected the p53 peptide exhibits only a small favorable contribution from Lennard-Jones interactions with the lid given the small number of contacts observed. However unfavorable Coulombic interactions with the lid contribute overall to a positive lid-p53 interaction energy profile. The situation differs for Nutlin-3a and Bzd that show more negative Lennard-Jones interaction energies owing to their extended contacts with the lid, and additionally the Bzd complex is further stabilized by significant Coulombic interactions owing the interactions of the carboxylic group of Bzd with Thr16 and Ser17 (Fig 8D2). By contrast Pip2 shows modest Lennard-Jones interactions with the lid, and unfavorable Coulombic interactions. Thus the enhanced affinity of Pip2 for lid including constructs does not arise from direct interactions between the ligand and the lid.

Fig 9B shows the changes in lid-core interaction energies for each complex. Binding of p53 TAD is associated with a significant loss of Lennard-Jones energy owing to the loss of several contacts between the lid and core regions. The same applies in the Nutlin-3a complex, but this time the loss of Lennard-Jones interactions is offset by favorable lid-core interactions, such that changes in lid-core energetics slightly favor complex formation. This is not the case for the Bzd complex where overall the lid-core interactions are destabilizing. In stark contrast, strong lid-core Coulombic interactions contribute favorably to the stabilization of the Pip2-MDM2 complex, and further breakdown into of the lid-core energetics indicate that the strongest pairwise contribution arise from the formed salt bridge between Glu23 and Arg97 (Fig 8E2).

Fig 9C depicts changes in lid-lid intramolecular non-bonded energetics. The contribution is always positive, indicating that ligand binding has introduced strain in the lid in all instances, but particularly so in the Nutlin-3a and Bzd complexes. The result may appear surprising for the Pip2 complex, since α-helix formation in the lid is associated with the formation of several hydrogen-bonds between the backbone of several lid residues in segment 16–24. However further breakdown of the lid-lid interaction energy profiles indicates that while the additional hydrogen-bonding interactions and several hydrophobic contacts in segment 16–24 do indeed contribute favorably to complex formation, this contribution is offset by a loss of intramolecular interactions in segment 1–15 that adopts a more extended conformation than seen in apo MDM2 (S5 Fig).

Overall summation of all components in Fig 9 for each ligand indicates that ligand binding is associated with favorable lid energetics only for Pip2 and a key stabilizing interactions can be traced to favorable lid-core Coulombic interactions between lid residues Glu23 and core residue Arg97.

## Discussion

### An ensemble-view of MDM2 lid-ligand interactions

Little is known about the conformational preferences of the MDM2 lid region owing to its considerable flexibility that hinders experimental studies, and a slow rate of exchange (>10 ms) between open and closed states that is inaccessible to conventional molecular simulations. Further, because the lid is highly flexible in its distinct closed or open states, an accurate characterization of lid interactions necessitates a description in terms of structural ensembles rather than a single representative structure. The present study addressed the technical challenge of computing atomically detailed lid structural ensembles with the aid of accelerated molecular dynamics, umbrella sampling and variational free energy profile methodologies. Altogether, over 10 microseconds of biased molecular dynamics simulations was used to generate free-energy landscapes for the MDM2 lid region in five different conditions. This likely represents the largest effort to date to resolve the conformational ensembles of the MDM2 lid region by means of explicit-solvent atomistic molecular dynamics simulations. The computed FES were deemed reasonably well converged (S2–S3 Figs), and the predicted free energy difference between open and closed lid states in apo MDM2 was in reasonable agreement with experimental data (within 1 kcal.mol^-1^). The shift to an open lid state in the MDM2-p53 peptide complex was observed, in expectation with previous NMR studies.[18] These observations give confidence in the accuracy of the computed lid ensembles for the small-molecule bound simulations, for which relatively little was known prior to this study.

### Evidence for multiple closed states of the MDM2 lid

A striking novel result from this study is that the MDM2 lid adopts different closed states when bound to different classes of small-molecules. In apo MDM2 the closed state of the lid lies mainly over helix α2, but in Nutlin-3a or Bzd bound simulations, the lid moves away from helix α2 to cover the small-molecule ligands. In Pip2 bound simulations, the base of the lid orders into a α-helix/β-turn motif, with the rest of the lid showing considerable disorder. Thus it is inadequate to picture the MDM2 lid in equilibrium between a well-defined open and closed lid state. Instead the flexibility of the lid enables considerable adjustments in the closed state to best accommodate chemically distinct ligands. Comparison of the structures sampled in the FES depicted in Fig 2 shows that the conformations the lid adopts when p53 is bound are broadly present in the apo FES. However, conformations similar to the major conformations seen in the presence of the small-molecule ligands were not detected in the apo ensemble. This suggests that significant induced-fit is necessary to fine-tune interactions between the small-molecule ligands and the lid region.

### The solubilizing groups of MDM2 antagonists are potential MDM2 lid-interacting groups

The Nutlin-3a and Bzd ligands were both found to establish significant interactions with the lid region (Figs 6C, 6D, 8C2 and 8D2). In the case of Nutlin-3a, significant hydrophobic contacts and hydrogen-bonding interactions involve lid residue Thr10 and the piperazinone carbonyl group. In the case of Bzd, the carboxylate moiety forms significant hydrogen-bonding interactions with lid residues Thr16 and Ser17. By comparison with the structural ensembles of apo-MDM2 and Pip2-bound MDM2, it can be seen that these additional interactions drag the lid further over these ligands. In the Pip2-MDM2 complex, the lid cannot easily access the Pip2 carboxylate moiety because it is partially covered by core residues His96 and Lys94. Successful MDM2 antagonists generally position hydrophobic moieties in the p53/MDM2 binding cleft owing to the relatively apolar character of this binding site. Acceptable solubility of MDM2 small-molecule antagonists typically requires the introduction of an additional solubilizing group. On the basis of structural models where the MDM2 lid was absent, solubilizing groups in many classes of MDM2 antagonists have generally been positioned to lie over the surface or away from the protein towards region of space that have been assumed to be occupied solely by solvent molecules [37]. Although, it has been known for a long time that the precise chemical nature of the solubilizing group can substantially modulate the binding affinity of MDM2 ligands, an explanation for this observation has been lacking. For instance, removal of the solubilizing group in Nutlin derivative RG7112 decreases binding affinity by a factor of 100, whereas in Bzd analogues, the length and acidic/basic nature of the solubilizing group can modulate binding affinity by a factor of 85 [37,38]. The present results suggest that MDM2 antagonist design programs should routinely consider the possibility that solubilizing groups may interact with the lid region and that this may significantly impact binding affinities.

### Structural basis for favorable Pip2-MDM2 lid interactions

The Nutlin-3a and Bzd ligands are known to show little gains in potency through lid interactions, yet significant interactions with a large portion of the lid region were observed (Fig 6C and 6D). Energetic analysis revealed that the favorable ligand-lid interactions are here offset by unfavorable lid-core and lid-lid interactions (Fig 9). By contrast, the potency of Pip2 ligands benefits significantly from the presence of the lid region, yet relatively fewer contacts between the ligand and the lid are observed (Fig 6E). This is corroborated by the energetic analysis (Fig 9) that revealed unfavorable lid-ligand and lid-lid interactions that are offset by favorable lid-core interactions. Additionally all Pip2-lid contacts occur at or after residue 14 in the lid primary sequence, whereas Nutlin-3a and Bzd ligands also engage with lid residues in segment 6–14, notably with their solubilizing groups. Interactions with this segment are associated with a significant local decrease in lid flexibility (Fig 3A, 3C and 3D). These observations suggest an entropy-enthalpy compensation mechanism is also at play; in other words Nutlin-3 and Bzd ligands do not benefit significantly from the additional contacts formed with the lid because those also involve residues that were significantly disordered in apo MDM2.

A significant difference in Pip2-bound simulations is that lid residue Glu23 forms a stable salt-bridge with Arg97 in the core of MDM2, while Gln24 is hydrogen bonded to backbone atoms of Pro20, Ile19 and Thr16. This network of hydrogen-bonding interactions effectively locks the base of the lid into the observed α-helix/β-turn motif (Fig 4E). However this pattern of interactions is not observed in the apo MDM2 simulations, suggesting that this conformation is only stable in the Pip2 complex because the *meta*-chlorophenyl ring of Pip2 also packs against lid residues 14–16 (Fig 8E2). Evidently Pip2 cannot engage directly with the lid region in short MDM2 constructs that are truncated at residue 17. Therefore the origin of the high affinity of Pip2 for the extended lid MDM2 construct of Michelsen et al. is attributed to the indirect stabilization of the lid α-helix at residues 21–24 *via* hydrophobic contacts between the *meta*-chlorophenyl ring of Pip2 and Val14/Thr16.

Interestingly, Bista et al. have recently reported that chloroindole carboxylate derivatives are also able to interact with the lid through conformational adjustments that fit a *para*-chlorobenzyloxy benzyl moiety deep near the α-helix lid [39]. Though the binding affinity data for these ligands was not reported for the MDM2 constructs studied here, this suggests that different strategies may be available to stabilize the α-helix lid region. Intriguingly, although absent in the Nutlin-3a and Bzd-bound MDM2 simulations, the α-helix lid has been observed in some crystallographic complexes of these ligands with shorter MDM2 (17–125) constructs [21,23]. However in the present simulations, lid residues 1–16 engage in interactions with the ligands and the MDM2 core region that preclude formation of the α-helix lid in segment 19–24. This is corroborated by additional (ca. 100 ns timescale) MD simulations of Bzd/Nutlin-3a bound to MDM2 1–119 and 17–125 that suggest the α-helix lid is only stable in the short construct (S6–S7 Figs).

Taken together, these observations suggest that a strategy to productively exploit ligand-lid interactions is to 1) stabilize with hydrophobic contacts a lid conformation where lid residues 14–16 packs against the α-helix lid, and 2) hinder undesirable lid interactions with residues 1–13 by positioning solubilizing groups to interact with ordered MDM2 core residues. In Pip2 this is achieved through hydrophobic contacts of the *meta*-chlorophenyl ring with Val14/Thr16 and hydrogen-bonding interactions of a carboxylate moiety with His96 and Lys94, but other solutions may be possible.

### Conclusion

In summary, a detailed analysis of the interactions of the N-terminal domain of MDM2 with several ligands was undertaken to elucidate conformational preferences of the MDM2 lid region and to rationalize the origin of the ca. 25-fold activity improvement of the Pip2 ligand for constructs including an extended lid region. The simulations of apo-MDM2 indicate that the lid is disordered, adopting a mixture of open and closed lid states. Binding of p53 shifts the equilibrium towards an open disordered state, in agreement with reported NMR data.[18] A novel significant finding is that the MDM2 lid exhibits different conformational preferences and significant interactions with different classes of small-molecule p53/MDM2 antagonists. Structural and energetic analyses show that the enhanced affinity of Pip2 for MDM2 constructs that include the full lid is due to hydrophobic contacts that facilitate structuring of an α-helix/β-turn motif in lid residues 17–24. Nutlin-3a or Bzd ligands that show similar affinity for short or long MDM2 lid constructs hinder formation of this motif because they also engage through their solubilizing groups segments of the lid earlier in the primary sequence that are more disordered in apo MDM2. Taken together, these findings suggest that a strategy to productively exploit MDM2 lid-interactions for inhibitor design is to target the base of the lid with deep hydrophobic contacts, and to position solubilizing groups so as to minimize the likelihood of interactions with polar lid residues and lid residues distant from the lid base. These findings may be of significance to facilitate the development of novel potent and selective p53/MDM2 ligands as putative anti-cancer agents, and more generally to suggest new hypotheses for productively targeting disordered protein regions in structure-based drug design efforts.

## Materials and Methods

### 1. Systems setup

#### 1.1 apo-MDM2

Five different structures (models 2, 3, 4, 10 and 13) from a NMR solution ensemble of the N-terminal domain of MDM2 (residues 1–119) (PDB ID: 1Z1M)[19] were selected as the initial conformations for preliminary MD simulations of MDM2 in the apo state. These states were selected to provide an initial broad coverage of plausible lid conformational states.

#### 1.2. MDM2 complexes

To build models of the different MDM2 complexes, the following PDB structures were used as templates: 1YCR (p53) [16], 4HG7 (Nutlin-3a) [21], 1T4E (Bzd) [23], and 4HBM (Pip2) [24]. Each complex was superimposed onto the apo NMR ensemble (model 4 for p53-MDM2, model 2 for the ligand-bound systems) such that each complex included a complete form of the lid (residues 1–119). Models were prepared with the *leap* utility from the AMBER12 suite,[40] using the ff99SBildnnmr force field [41].

The parameters of each ligand were generated using the *antechamber* utility from the AMBER12 suite of programs [40], in combination with the general AMBER force field (GAFF).[42] Atomic charges were assigned using the AM1-BCC method [43,44]. Each protein or protein-ligand complex was immersed in a cubic periodic box of TIP3P water molecules [45], (6600 to 6800 water molecules depending on the system) and neutralized by addition of the appropriate number of Cl^-^ ions.[46] This was followed by steepest-descent energy minimization to avoid steric clashes. To facilitate sampling of extended lid conformations the box edges were located at least 15 Å away from the surface of the protein.

### 2. Molecular dynamics simulation protocols

The bulk of computational studies of MDM2 ligand interactions have neglected the lid,[47–56] Notable exceptions include work from Verkhivker and Dastidar et al. that have studied lid conformations over ca. 10 ns time scales [57,58]. However work from Showalter et al. suggested that the lid exchanges between closed and open states over a much slower (>10 ms) timescale [18]. To overcome this technical challenge, a protocol featuring use of aMD [27–29], US [30], and vFEP [31,32] was adopted.

#### 2.1. Preliminary molecular dynamics simulations

Preliminary conventional MD (cMD) simulations were performed to optimise aMD simulation protocols. All simulations were performed using the *pmemd* module of AMBER12. The cut-off distance for the non-bonded interactions was 10 Å and periodic boundary conditions were used. The particle mesh Ewald method was used to treat long-range electrostatic interactions [59]. The SHAKE algorithm was applied to all bonds involving hydrogens and an integration step of 2.0 fs was used throughout [60]. Each system was heated to 300 K over a 50 ps interval using a weak coupling algorithm [61], with the solute atoms restrained with positional restraints of 50.0 kcal mol^-1^ Å^-2^. Then a 200 ps equilibration was performed to allow the solvent to redistribute around the positionally restrained solute. The system was subsequently allowed to evolve freely unrestrained at constant temperature (300 K) and pressure (1 atm) using the weak-coupling algorithm [61]. System coordinates were collected every 20 ps for further analysis. Five cMD simulations of apo-MDM2 were performed starting from different snapshots corresponding to Models 2, 3, 4, 10 and 13 from the NMR solution ensemble of apo-MDM2 [19]. The total simulation time for apo-MDM2 was 750 ns. For the small-molecule MDM2 complexes, 100 (Nutlin3a, Bzd) to 200 (Pip2) ns of unrestrained cMD were performed. The p53-MDM2 complex was simulated for 100 ns. Two additional 100 ns cMD of Nutlin3a-MDM2 and Bzd-MDM2 were performed using as starting points representative conformations from the lowest free energy bins. Two more 100 ns cMD of Nutlin-3a-MDM2 and Bzd-MDM2 were performed using a truncated form of the lid (17–125) with a previously the truncated lid adopting a α-helix at residues 20–24. The N- terminus of this truncated lid was capped with acetyl (ACE).

#### 2.2. Accelerated molecular dynamics (aMD)

aMD adds a positive boost energy to the potential energy function, effectively reducing the height of energetic barriers and enhancing conformational sampling [29,62,63]. Unlike many other enhanced sampling methods such as metadynamics [64,65], aMD does not require a prior definition of a collective variable to enhance sampling. A boost potential, *ΔV(* ***r*** *)*, is applied when the average potential energy of the system, *V(* ***r*** *)*, is below a previously defined reference potential energy *E*_*p*_. The modified potential used for MD simulations, V*( ***r*** *)*, is then given by eq 1 [29,66]:

$$V*(r)=\{\begin{array}{c} V(r)\\ V(r)+\Delta V(r) \end{array}\;\begin{array}{c} V(r)>E_{P}\\ V(r)<E_{P} \end{array}$$ (1)

A dual boost protocol was used (eq 2), with a boost potential energy applied to all the atoms in the system with an extra dihedral boost to the torsions (using *iamd = 3* keyword in AMBER 12):

$$\Delta V(r)=\frac{{\left(E_{P}-V(r)\right)}^{2}}{\left(\alpha_{P}+E_{P}-V(r)\right)}+\frac{{\left(E_{D}-V_{D}(r)\right)}^{2}}{\left(\alpha_{D}+E_{D}-V_{D}(r)\right)}$$ (2)

where *V*_*D*_ is the dihedral energy, *E*_*P*_ and *E*_*D*_ are the reference potential and dihedral energies and *α*_*P*_ and *α*_*D*_ are the acceleration parameters that describe the strength of the boost for each term. To focus enhanced sampling on the lid conformations, positional restraints were applied to the MDM2 core domain (residues 30 to 119) and the ligands (20 kcal mol^-1^ Å^-2^) whereas residues 1 to 30 were allowed to evolve freely. Those restrictions were subsequently removed prior to the beginning of the US calculations. The chosen aMD parameters (*E*_*P*_, *α*_*P*_, *E*_*D*_, *α*_*D*_) were initially set according to guidelines from previous work [67,68], and were subsequently tested and modified until an enhancement in the sampling of lid conformations was achieved. The final parameters are given in Table 1.

**Table 1:** aMD parameters used in the present simulations.

System	E_P_	α_P_	E_D_	α_D_
**Apo-MDM2**	-125,306	10,000	3,000	83
**p53-MDM2**	-155,742	10,000	3,000	83
**Nutlin3a-MDM2**	-142,371	10,000	3,000	83
**Bzd-MDM2**	-150,118	10,000	3,000	83
**Pip2-MDM2**	-133,000	10,000	3,000	83

The choice of the parameters was initially done using according to guidelines from Bucher et al. [68] and Pierce et al. [67], and further adjustments were done with the help of preliminary cMD simulations. All the parameters are given in kcal.mol^-1^.

aMD simulations were performed using the same conditions described for the cMD simulation protocols, and were initiated from an equilibrated semi-closed conformation (Model 2). The aMD simulations provided significantly enhanced lid sampling compared to cMD simulations (S1 Fig), e.g. multiple transitions between open and closed states were observed in apo-MDM2 (S1 Movie in S3 Dataset,), whereas no transitions were observed in any apo MDM2 cMD simulation. Additionally increased conformational fluctuations were observed for ligand-bound aMD simulations (S2 Movie in S3 Dataset,). Equilibrium properties of the unbiased potential can in principle be recovered by reweighting statistics with the exponential of the boost potential, *exp*(*βΔV(* ***r*** *)*) [29]. In practice, especially for large biomolecular systems, a useful level of enhanced sampling is only reached if large values of the boost potentials are used, which usually leads to unacceptable variance in the reweighted results.[69,70] Different forms of aMD have been tested to overcome these limitations although no methodology has yet been successfully applied to large proteins [71–73]. Another drawback of the aMD methodology is that, in common with many enhanced sampling methodologies, it is difficult to recover information about the kinetics of the sampled processes [74].

#### 2.3 Umbrella sampling (US) calculations

**Fig 10:**
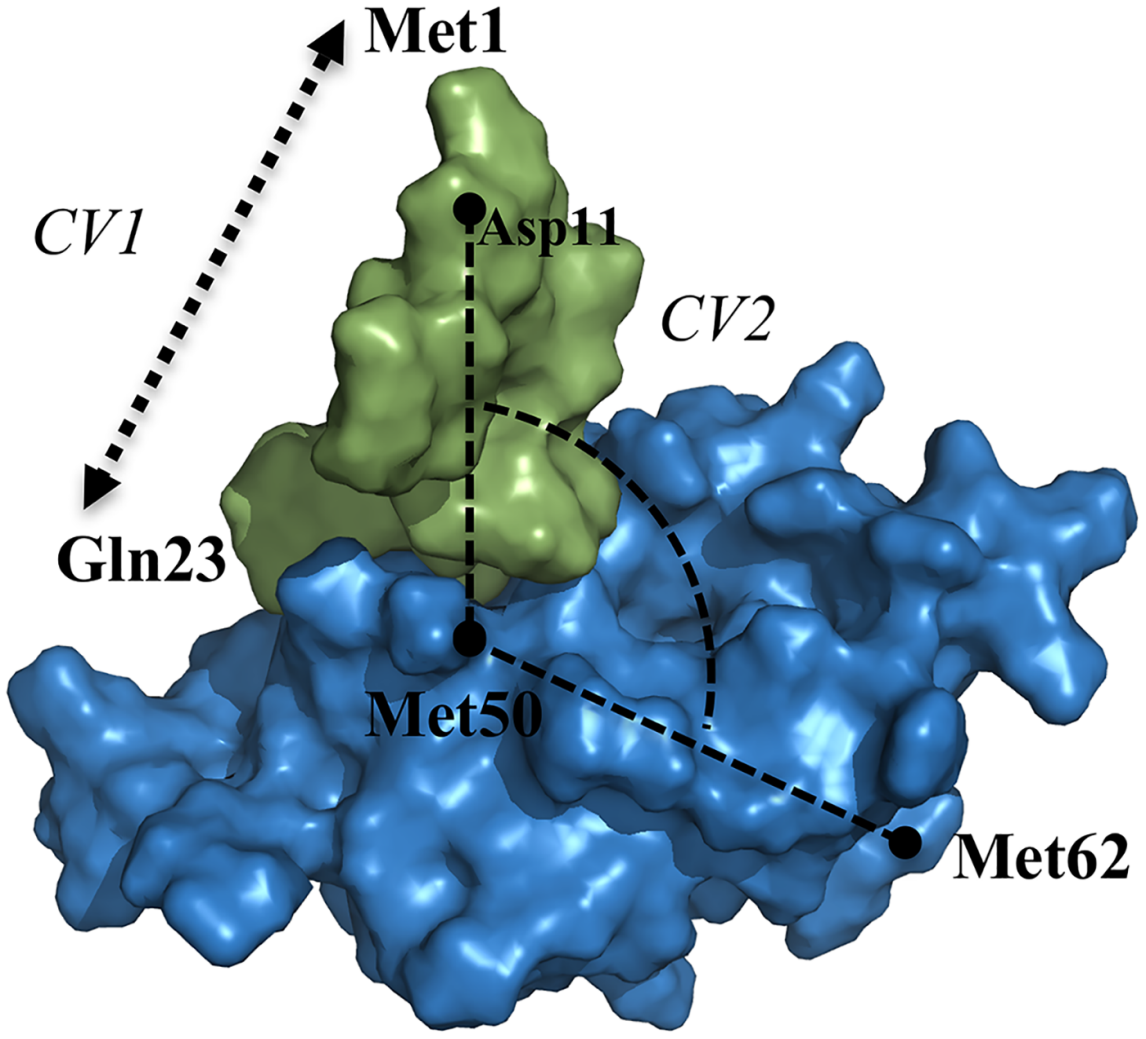
Definition of the collective variables used during the US simulations of the MDM2 lid dynamics. CV1: lid extension (Met1(Cα)-Glu23(Cα) distance); CV2: lid-core angle (Met62(Cα)-Met50(Cα)-Asp11(Cα).

By means of the above aMD protocol, a broad range of lid conformations were obtained for apo and bound MDM2 complexes. Two suitable collective variables were selected to discriminate between the different observed lid states: CV1 defines the extension of the lid as a function of the distance between the alpha carbons of residues 1 and 23. CV2 is the lid-core angle, i.e. the angle between the alpha carbons of residues 11, 50 and 62 (Fig 10).

Such a choice allows the identification of the different states of the lid: the p53-binding site is occluded by the lid (“closed” conformation) when the lid-core angle is below 80° and the lid extension is larger than 24 Å. Other values lead to a solvent-exposed p53-binding site pocket (lid “opened”).

Next, an US protocol was applied to compute an equilibrium distribution for each lid ensemble. CV1 was sampled from 3 to 49 Å with a 2 Å interval and CV2 from 20 to 140° (158° for p53 complex) with a 6° interval reaching a total of 504 windows for each system (576 for p53 complex). No restraints on the MDM2 core region or the ligand were applied. The initial coordinates for every window were taken from the closest snapshot sampled during the aMD simulation. A similar strategy has been used successfully by Ostmeyer et al. to seed independent US simulations with relaxed conformations from long-time MD simulations [75]. For each simulation bin, 500 ps simulation were considered to re-equilibrate the system before running 4 ns in the production phase. The same conditions as in the cMD simulations were applied and in addition a harmonic potential restraint of 1 kcal mol^-1^Å^-2^ and 0.12 kcal mol^-1^deg^-2^ was used for CV1 and CV2 respectively. The force constants of the restraining potentials were chosen to avoid excessive energetic penalties for conformations deviating slightly from the target CV values as this could otherwise hinder conformational sampling of the other degrees of freedoms of the system during the US simulations. This risk was also lessened because relatively loose collective variables were used in this study (i.e. there are several ways for the lid to adopt a given extension/orientation as defined by CV1 and CV2). Values of the reaction coordinates were stored every 10 fs for post-processing.

#### 2.4. vFEP reweighting

The 2D variational Free Energy Profile (vFEP) method [31,32] was used to obtain unbiased free energy profiles along the two collective variables. vFEP is a maximum likelihood parametric approach to reweight biased simulation data. For smoothly varying free energy surfaces, the method has been shown to yield converged FES with fewer windows and with a fraction of the statistics required with WHAM [76]. To estimate uncertainties in bin free energies, all US trajectories were sub-divided into four parts of equal duration and analysed separately (S2–S3 Figs). The 2D FES projection lumps together local minima and no attempt was made to recover an estimate of the kinetics of the open/close lid transitions from this projection.

### 3. Analysis of the molecular dynamics trajectories

Three-dimensional structures and trajectories were visually inspected using the computer graphics programs PyMOL[77] and VMD [78]. Hydrogen bonds and hydrophobic contacts between the MDM2 lid and MDM2 core/ligand regions were monitored using *ptraj* and *cpptraj* modules in AmberTools 12 [40,79]. The formation of a hydrogen bond was considered when the distance between donor and acceptor was shorter or equal to 3.0 Å and the angle between the acceptor, hydrogen and donor atoms was equal or larger than 154°. A hydrophobic contact was defined when the distance between two carbon atoms was less than 5 Å. To avoid counting a large number of trivial contacts, for every lid residue *i*, intramolecular lid-lid hydrophobic contacts with immediate neighboring residues (*i*+1, *i*-1) were excluded from the analysis.

Diverse ensemble-averages of lid-residue properties were computed, including the average number of hydrogen bonds with core/ligand atoms, average hydrophobic contacts with core/ligand atoms, average backbone heavy-atom RMSD to a representative conformation taken from the lowest free energy bin (*RMSD*_*i*_) and average helical (*H*_*i*_), sheet (*S*_*i*_) turn (*T*_*i*_) or coil (*C*_*i*_) propensity, the latter four computed according to the DSSP code [35,36]. Lid energetics were also characterized by component analysis of interaction energies using the mmpbsa.py software [80]. Specific quantities evaluated for each complex were: ligand-lid interaction energies (*E*_*lig-lid*_), lid-core interaction energies (*E*_*lid-core*_), and lid-lid intramolecular non-bonded energies (*E*_*lid-lid*_).

All observables and quantities were obtained by reweighting statistics from the US sampled snapshots according to eq 3.

$$\langle A_{i}\rangle =\frac{\sum_{j=1}^{N}(\frac{1}{M}\sum_{k=1}^{M}A_{j,k})\cdot e^{-\beta \Delta G_{j}}}{\sum_{j=1}^{N}e^{-\beta \Delta G_{j}}}$$ (3)

where <*A*_*i*_> is the ensemble average of the property of interest for lid residue *i*, *N* is the number of US bins, *M* is the number of snapshots in bin *j*, *A*_*j*,*k*_ is the value of property *A*_*i*_ for snapshot *k* in bin *j*, and *ΔG*_*j*_ is the free energy of bin *j* obtained by vFEP reweighting. To estimate uncertainties in the computed properties, the simulation data was split in four consecutive blocks of 1 ns each and property values computed separately. Mean <*A*_*i*_> values are reported along with one standard error. Lastly, graphical depictions of representative lid structural ensembles for each system were obtained by randomly sampling 20 snapshots from the pooled US snapshots according to their computed equilibrium properties.

## Supporting Information

S1 Fig MDM2 lid distribution plot (in number of structures) projected on X: lid extension, in Å and Y: lid-core angle, in degrees. Top: Apo-MDM2, cMD (left) and aMD (right). Bottom. Nutlin3a-MDM2, cMD (left) and aMD (right).
(TIFF)

S2 Fig Free energy surfaces for the MDM2 lid conformational changes projected on CV1 (lid extension, in Å) and CV2 (lid-core angle, in degrees) in different time windows. Energies are in kcal.mol^-1^. (Left) apo-MDM2 simulations. (Right) p53-MDM2 simulations.
(TIFF)

S3 Fig Free energy surfaces for the MDM2 lid conformational changes projected on CV1 (lid extension, in Å) and CV2 (lid-core angle, in degrees) in different time windows. Energies are in kcal.mol^-1^. (Left) Nutlin3a-MDM2 simulations. (Middle) Bdz-MDM2 simulations. (Right) Pip2-MDM2 simulations.
(TIFF)

S4 Fig Ligand-dependent modulation of MDM2 lid flexibility. Per-lid residue average backbone RMSD in Å considering heavy atoms. A) apo, B) p53, C) Nutlin-3a, D) Bzd, E) Pip2.
(TIFF)

S5 Fig Changes in lid-lid interaction energy upon Pip2 binding for lid segments 16–24 and lig segments 1–24. Lennard-Jones energies are depicted in red, Coulombic energies in blue and total interaction energies in gray. Energies are in kcal.mol^-1^.
(TIFF)

S6 Fig Secondary structure propensity of Pro20-Gln24 lid residues along cMD simulations of Nutlin3a-MDM2 complex. A) cMD simulations using as starting point a representative conformation from the minimum free energy well of the US of Nutlin3a-MDM2 complex (no α-helix displayed) (Residues 1–119). B) cMD simulations using as starting point a truncated form of the lid (Residues 17–25) starting from a previously formed α-helix between residues Pro20 and Gln24. Red: helix; Green: Turn; Grey: Coil.
(TIFF)

S7 Fig Secondary structure propensity of Pro20-Gln24 lid residues along cMD simulations of Bzd-MDM2 complex. A) cMD simulations using as starting point a representative conformation from the minimum free energy well of the US of Bzd-MDM2 complex (no α-helix displayed) (Residues 1–119). B) cMD simulations using as starting point a truncated form of the lid (Residues 17–25) starting from a previously formed α-helix between residues Pro20 and Gln24. Red: helix; Green: Turn; Grey: Coil.
(TIFF)

S1 Dataset pdbs_SI.zip: PDB files corresponding to fig: 7A2, 7A3, 7B2, 7C2, 7D2 and 7E2. (ZIP)

S2 Dataset inputs_SI.zip: Sample input files for the aMD or US simulations. (ZIP)

S3 Dataset Movies.zip: Sample trajectories from representative aMD simulations of apo MDM2 (S1 Movie) or Bzd-bound MDM2 (S2 Movie). (ZIP)

## References

[1] Habchi J, Tompa P, Longhi S, Uversky VN (2014) Introducing protein intrinsic disorder. Chem Rev 114: 6561–6588. 10.1021/cr400514h 24739139

[2] Uversky VN (2011) Intrinsically disordered proteins from A to Z. Int J Biochem Cell Biol 43: 1090–1103. 10.1016/j.biocel.2011.04.001 21501695

[3] Dyson HJ, Wright PE (2005) Intrinsically unstructured proteins and their functions. Nat Rev Mol Cell Biol 6: 197–208. 10.1038/nrm1589 15738986

[4] Whitford PC, Sanbonmatsu KY, Onuchic JN (2012) Biomolecular dynamics: order-disorder transitions and energy landscapes. Rep Prog Phys 75: 076601 10.1088/0034-4885/75/7/076601 22790780 PMC3695400

[5] Cuchillo R, Michel J (2012) Mechanisms of small-molecule binding to intrinsically disordered proteins. Biochem Soc Trans 40: 1004–1008. 10.1042/BST20120086 22988855

[6] Hammoudeh DI, Follis AV, Prochownik EV, Metallo SJ (2009) Multiple Independent Binding Sites for Small-Molecule Inhibitors on the Oncoprotein c-Myc. J Am Chem Soc 131: 7390–7401. 10.1021/ja900616b 19432426

[7] Michel J, Cuchillo R (2012) The impact of small molecule binding on the energy landscape of the intrinsically disordered protein C-myc. PLoS ONE 7: e41070 10.1371/journal.pone.0041070 22815918 PMC3397933

[8] Herbert C, Schieborr U, Saxena K, Juraszek J, De Smet F, et al (2013) Molecular mechanism of SSR128129E, an extracellularly acting, small-molecule, allosteric inhibitor of FGF receptor signaling. Cancer Cell 23: 489–501. 10.1016/j.ccr.2013.02.018 23597563

[9] Fenton AW (2013) Are all regions of folded proteins that undergo ligand-dependent order-disorder transitions targets for allosteric peptide mimetics? Biopolymers 100: 553–557. 10.1002/bip.22239 23520021 PMC3735636

[10] Michel J (2014) Current and emerging opportunities for molecular simulations in structure-based drug design. Phys Chem Chem Phys 16: 4465–4477. 10.1039/c3cp54164a 24469595 PMC4256725

[11] Brown CJ, Lain S, Verma CS, Fersht AR, Lane DP (2009) Awakening guardian angels: drugging the p53 pathway. Nat Rev Cancer 9: 862–873. 10.1038/nrc2763 19935675

[12] Brown CJ, Cheok CF, Verma CS, Lane DP (2011) Reactivation of p53: from peptides to small molecules. Trends Pharmacol Sci 32: 53–62. 10.1016/j.tips.2010.11.004 21145600

[13] Römer L, Klein C, Dehner A, Kessler H, Buchner J (2006) p53—A Natural Cancer Killer: Structural Insights and Therapeutic Concepts. Angew Chem Int Ed 45: 6440–6460. 10.1002/anie.200600611 16983711

[14] Honda R, Tanaka H, Yasuda H (1997) Oncoprotein MDM2 is a ubiquitin ligase E3 for tumor suppressor p53. FEBS Lett 420: 25–27. 9450543 10.1016/s0014-5793(97)01480-4

[15] Momand J, Zambetti GP, Olson DC, George D, Levine AJ (1992) The mdm-2 oncogene product forms a complex with the p53 protein and inhibits p53-mediated transactivation. Cell 69: 1237–1245. 10.1016/0092-8674(92)90644-R 1535557

[16] Kussie PH, Gorina S, Marechal V, Elenbaas B, Moreau J, et al (1996) Structure of the MDM2 oncoprotein bound to the p53 tumor suppressor transactivation domain. Science 274: 948–953. 8875929 10.1126/science.274.5289.948

[17] Oliner JD, Pietenpol JA, Thiagalingam S, Gyuris J, Kinzler KW, et al (1993) Oncoprotein MDM2 conceals the activation domain of tumour suppressor p53. Nature 362: 857–860. 10.1038/362857a0 8479525

[18] Showalter SA, Bruschweiler-Li L, Johnson E, Zhang F, Brüschweiler R (2008) Quantitative Lid Dynamics of MDM2 Reveals Differential Ligand Binding Modes of the p53-Binding Cleft. J Am Chem Soc 130: 6472–6478. 10.1021/ja800201j 18435534

[19] Uhrinova S, Uhrin D, Powers H, Watt K, Zheleva D, et al (2005) Structure of free MDM2 N-terminal domain reveals conformational adjustments that accompany p53-binding. J Mol Biol 350: 587–598. 15953616 10.1016/j.jmb.2005.05.010

[20] McCoy MA, Gesell JJ, Senior MM, Wyss DF (2003) Flexible lid to the p53-binding domain of human Mdm2: Implications for p53 regulation. Proc Natl Acad Sci USA 100: 1645–1648. Available: http://eutils.ncbi.nlm.nih.gov/entrez/eutils/elink.fcgi?dbfrom=pubmed&id=12552135&retmode=ref&cmd=prlinks. 12552135 10.1073/pnas.0334477100 PMC149886

[21] Anil B, Riedinger C, Endicott JA, Noble MEM (2013) The structure of an MDM2–Nutlin-3a complex solved by the use of a validated MDM2 surface-entropy reduction mutant. Acta Cryst 69: 1358–1366. 10.1107/S0907444913004459 23897459

[22] Vassilev LT (2004) In Vivo Activation of the p53 Pathway by Small-Molecule Antagonists of MDM2. Science 303: 844–848. 10.1126/science.1092472 14704432

[23] Grasberger BL, Lu T, Schubert C, Parks DJ, Carver TE, et al (2005) Discovery and cocrystal structure of benzodiazepinedione HDM2 antagonists that activate p53 in cells. J Med Chem 48: 909–912. 15715460 10.1021/jm049137g

[24] Michelsen K, Jordan JB, Lewis J, Long AM, Yang E, et al (2012) Ordering of the N-Terminus of Human MDM2 by Small Molecule Inhibitors. J Am Chem Soc 134: 17059–17067. 10.1021/ja305839b 22991965

[25] Hoe KK, Verma CS, Lane DP (2014) Drugging the p53 pathway: understanding the route to clinical efficacy. Nat Rev Drug Discov 13: 217–236. 10.1038/nrd4236 24577402

[26] Shangary S, Wang S (2009) Small-Molecule Inhibitors of the MDM2-p53 Protein-Protein Interaction to Reactivate p53 Function: A Novel Approach for Cancer Therapy. Annu Rev Pharmacol Toxicol 49: 223–241. 10.1146/annurev.pharmtox.48.113006.094723 18834305 PMC2676449

[27] Pierce LCT, Salomon-Ferrer R, Augusto F de Oliveira C, McCammon JA, Walker RC (2012) Routine Access to Millisecond Time Scale Events with Accelerated Molecular Dynamics. J Chem Theory Comput 114: 2997–3002. 10.1021/ct300284c PMC3438784 22984356

[28] Markwick PRL, Pierce LCT, Goodin DB, McCammon JA (2011) Adaptive Accelerated Molecular Dynamics (Ad-AMD) Revealing the Molecular Plasticity of P450cam. J Phys Chem Lett 2: 158–164. 10.1021/jz101462n 21307966 PMC3034398

[29] Hamelberg D, Mongan J, McCammon JA (2004) Accelerated molecular dynamics: a promising and efficient simulation method for biomolecules. J Chem Phys 120: 11919–11929. 15268227 10.1063/1.1755656

[30] Torrie GM, Valleau JP (1977) Nonphysical sampling distributions in Monte Carlo free-energy estimation: Umbrella sampling. J Comput Phys 23: 187–199. 10.1016/0021-9991(77)90121-8

[31] Lee TS, Radak BK, Huang M, Wong K-Y, York DM (2014) Roadmaps through Free Energy Landscapes Calculated Using the Multidimensional vFEP Approach. J Chem Theory Comput 10: 24–34. 24505217 10.1021/ct400691f PMC3912246

[32] Lee TS, Radak BK, Pabis A, York DM (2013) A New Maximum Likelihood Approach for Free Energy Profile Construction from Molecular Simulations. J Chem Theory Comput 9: 153–164. 10.1021/ct300703z 23457427 PMC3580863

[33] Best RB, Mittal J (2011) Free-energy landscape of the GB1 hairpin in all-atom explicit solvent simulations with different force fields: Similarities and differences. Proteins 79: 1318–1328. 10.1002/prot.22972 21322056 PMC4228318

[34] Best RB (2012) Atomistic molecular simulations of protein folding. Curr Opin Struct Biol 22: 52–61. 10.1016/j.sbi.2011.12.001 22257762

[35] Joosten RP, Beek te TAH, Krieger E, Hekkelman ML, Hooft RWW, et al (2011) A series of PDB related databases for everyday needs. Nucleic Acids Res 39: D411–D419. 10.1093/nar/gkq1105 21071423 PMC3013697

[36] Kabsch W, Sander C (1983) Dictionary of protein secondary structure: Pattern recognition of hydrogen-bonded and geometrical features—Kabsch—2004—Biopolymers—Wiley Online Library. Biopolymers 22: 2577–2637. 6667333 10.1002/bip.360221211

[37] Parks DJ, LaFrance LV, Calvo RR, Milkiewicz KL, José Marugán J, et al (2006) Enhanced pharmacokinetic properties of 1,4-benzodiazepine-2,5-dione antagonists of the HDM2-p53 protein–protein interaction through structure-based drug design. Bioorg Med Chem Lett 16: 3310–3314. 10.1016/j.bmcl.2006.03.055 16600594

[38] Fry DC, Wartchow C, Graves B, Janson C, Lukacs C, et al (2013) Deconstruction of a nutlin: dissecting the binding determinants of a potent protein-protein interaction inhibitor. ACS Med Chem Lett 4: 660–665. 10.1021/ml400062c 24900726 PMC4027557

[39] Bista M, Wolf S, Khoury K, Kowalska K, Huang Y, et al (2013) Transient protein states in designing inhibitors of the MDM2-p53 interaction. Structure 21: 2143–2151. 10.1016/j.str.2013.09.006 24207125 PMC4104591

[40] Case DA, Darden TA, Cheatham TE 3, Simmerling C, Wang J, et al (2012) AMBER12 Amber 12; University of California, San Francisco.

[41] Li DW, Brüschweiler R (2010) NMR-based protein potentials. Angew Chem Int Ed 49: 6778–6780. 10.1002/anie.201001898 20715028

[42] Wang J, Wolf RM, Caldwell JW, Kollman PA, Case DA (2004) Development and testing of a general amber force field. J Comput Chem 26: 114–114. 10.1002/jcc.20035 15116359

[43] Jakalian A, Bush BL, Jack DB (2000) Fast, efficient generation of high-quality atomic charges. AM1-BCC model: I. Method. J Comput Chem 21: 132–146. 10.1002/jcc.10128 12395429

[44] Jakalian A, Jack DB, Bayly CI (2002) Fast, efficient generation of high-quality atomic charges. AM1-BCC model: II. Parameterization and validation. J Comput Chem 23: 1623–1641. 12395429 10.1002/jcc.10128

[45] Jorgensen WL, Chandrasekhar J, Madura JD, Impey RW, Klein ML (1983) Comparison of simple potential functions for simulating liquid water. J Chem Phys 79: 926–935.

[46] Aaqvist J (1990) Ion-water interaction potentials derived from free energy perturbation simulations. J Phys Chem B 94: 8021–8024. 10.1021/j100384a009

[47] Niu RJ, Zheng Q-C, Zhang J-L, Zhang H-X (2013) Molecular dynamics simulations studies and free energy analysis on inhibitors of MDM2–p53 interaction. J Mol Graph Model 46: 132–139. 10.1016/j.jmgm.2013.10.005 24211465

[48] Almerico AM, Tutone M, Pantano L, Lauria A (2012) Molecular dynamics studies on Mdm2 complexes: An analysis of the inhibitor influence. Biochem Biophys Res Commun 424: 341–347. 10.1016/j.bbrc.2012.06.138 22771796

[49] Chen J, Wang J, Xu B, Zhu W, Li G (2011) Insight into mechanism of small molecule inhibitors of the MDM2–p53 interaction: Molecular dynamics simulation and free energy analysis. J Mol Graph Model 30: 46–53. 10.1016/j.jmgm.2011.06.003 21764342

[50] Joseph TL, Madhumalar A, Brown CJ, Lane DP, Verma CS (2010) Differential binding of p53 and nutlin to MDM2 and MDMX: Computational studies. Cell Cycle 9: 1167–1181. 10.4161/cc.9.6.11067 20190571

[51] Michel J, Harker EA, Tirado-Rives J, Jorgensen WL, Schepartz A (2009) In Silico Improvement of β 3-Peptide Inhibitors of p53•hDM2 and p53•hDMX. J Am Chem Soc 131: 6356–6357. 10.1021/ja901478e 19415930 PMC2754742

[52] Dastidar SG, Lane DP, Verma CS (2009) Modulation of p53 binding to MDM2: computational studies reveal important roles of Tyr100. BMC Bioinformatics 10 Suppl 15: S6 10.1186/1471-2105-10-S15-S6 19958516 PMC2788357

[53] Lee HJ, Srinivasan D, Coomber D, Lane DP, Verma CS (2007) Modulation of the p53-MDM2 Interaction by Phosphorylation of Thr18: A Computational Study. Cell Cycle 6: 2604–2611. 10.4161/cc.6.21.4923 17957142

[54] Espinoza-Fonseca LM, Trujillo-Ferrara JG (2006) Conformational changes of the p53-binding cleft of MDM2 revealed by molecular dynamics simulations. Biopolymers 83: 365–373. 10.1002/bip.20566 16817233

[55] Espinoza-Fonseca LM, Trujillo-Ferrara JG (2006) Transient stability of the helical pattern of region F19-L22 of the N-terminal domain of p53: a molecular dynamics simulation study. Biochem Biophys Res Commun 343: 110–116. 10.1016/j.bbrc.2006.02.129 16530164

[56] Zhong H, Carlson HA (2004) Computational studies and peptidomimetic design for the human p53-MDM2 complex. Proteins 58: 222–234. 10.1002/prot.20275 15505803

[57] Verkhivker GM (2012) Simulating Molecular Mechanisms of the MDM2-Mediated Regulatory Interactions: A Conformational Selection Model of the MDM2 Lid Dynamics. PLoS ONE 7: e40897 10.1371/journal.pone.0040897 22815859 PMC3397965

[58] Dastidar SG, Raghunathan D, Nicholson J, Hupp TR, Lane DP, et al (2011) Chemical states of the N-terminal “lid” of MDM2 regulate p53 binding: Simulations reveal complexities of modulation. Cell Cycle 10: 82–89. 10.4161/cc.10.1.14345 21191186

[59] Darden T, York D, Pedersen L (1993) Particle mesh Ewald: An N⋅log(N) method for Ewald sums in large systems. J Chem Phys 98: 10089–10092.

[60] Ryckaert J-P, Ciccotti G, Berendsen HJC (1977) Numerical integration of the cartesian equations of motion of a system with constraints: molecular dynamics of n-alkanes. J Comput Phys 23: 327–341.

[61] Berendsen HJC, Postma JPM, van Gunsteren WF, DiNola A, Haak JR (1984) Molecular dynamics with coupling to an external bath. J Chem Phys 81: 3684–3690. 10.1063/1.448118

[62] Markwick PRL, McCammon JA (2011) Studying functional dynamics in bio-molecules using accelerated molecular dynamics. Phys Chem Chem Phys 13: 20053–20065. 10.1039/c1cp22100k 22015376

[63] Hamelberg D, de Oliveira CAF, McCammon JA (2007) Sampling of slow diffusive conformational transitions with accelerated molecular dynamics. J Chem Phys 127: 155102–155102–9. 17949218 10.1063/1.2789432

[64] Iannuzzi M, Laio A, Parrinello M (2003) Efficient Exploration of Reactive Potential Energy Surfaces Using Car-Parrinello Molecular Dynamics. Phys Rev Lett 90: 238302 10.1103/PhysRevLett.90.238302 12857293

[65] Laio A, Parrinello M (2002) Escaping free-energy minima. Proc Natl Acad Sci USA 99: 12562–12566. 10.1073/pnas.202427399 12271136 PMC130499

[66] Wereszczynski J, McCammon JA (2012) Accelerated molecular dynamics in computational drug design. Methods Mol Biol 819: 515–524. 10.1007/978-1-61779-465-0_30 22183555

[67] Pierce L, Salomon-Ferrer R (2012) Routine Access to Millisecond Time Scale Events with Accelerated Molecular Dynamics—Journal of Chemical Theory and Computation (ACS Publications). J Chem Theory Comput 8: 2997–3002. 22984356 10.1021/ct300284c PMC3438784

[68] Bucher D, Grant BJ, Markwick PR, McCammon JA (2011) Accessing a Hidden Conformation of the Maltose Binding Protein Using Accelerated Molecular Dynamics. PLoS Comput Biol 7: e1002034 10.1371/journal.pcbi.1002034 21533070 PMC3080849

[69] Wang Y, McCammon JA (2012) Accelerated molecular dynamics: Theory, implementation and applications. AIP Conf Proc 1456: 165–172.

[70] Shen T, Hamelberg D (2008) A statistical analysis of the precision of reweighting-based simulations. J Chem Phys 129: 034103 10.1063/1.2944250 18647012

[71] Sinko W, de Oliveira CAF, Pierce LCT, McCammon JA (2012) Protecting High Energy Barriers: A New Equation to Regulate Boost Energy in Accelerated Molecular Dynamics Simulations. J Chem Theory Comput 8: 17–23. 10.1021/ct200615k 22241967 PMC3254191

[72] Wereszczynski J, McCammon JA (2010) Using Selectively Applied Accelerated Molecular Dynamics to Enhance Free Energy Calculations. J Chem Theory Comput 6: 3285–3292. 10.1021/ct100322t 21072329 PMC2976571

[73] Fajer M, Hamelberg D, McCammon JA (2008) Replica-Exchange Accelerated Molecular Dynamics (REXAMD) Applied to Thermodynamic Integration. J Chem Theory Comput 4: 1565–1569. 10.1021/ct800250m 19461870 PMC2651661

[74] Doshi U, Hamelberg D (2015) Towards fast, rigorous and efficient conformational sampling of biomolecules: Advances in accelerated molecular dynamics. Biochim Biophys Acta 1850: 878–888. Available: http://eutils.ncbi.nlm.nih.gov/entrez/eutils/elink.fcgi?dbfrom=pubmed&id=25153688&retmode=ref&cmd=prlinks. 10.1016/j.bbagen.2014.08.003 25153688

[75] Ostmeyer J, Chakrapani S, Pan AC, Perozo E, Roux B (2013) Recovery from slow inactivation in K+ channels is controlled by water molecules. Nature 501: 121–124. 10.1038/nature12395 23892782 PMC3799803

[76] Kumar S, Rosenberg JM, Bouzida D, Swendsen RH, Kollman PA (1992) The weighted histogram analysis method for free-energy calculations on biomolecules. I. The method. J Comput Chem 13: 1011–1021. 10.1002/jcc.540130812

[77] Delano WL (2002) The PyMOL Molecular Graphics System The PyMOL Molecular Graphics System, Version 1504; Schrödinger: Portland, OR, USA.

[78] Humphrey W, Dalke A, Schulten K (1996) VMD: Visual molecular dynamics. J Mol Graphics 14: 33–38. 8744570 10.1016/0263-7855(96)00018-5

[79] Roe DR, Cheatham TE III (2013) PTRAJ and CPPTRAJ: Software for Processing and Analysis of Molecular Dynamics Trajectory Data. J Chem Theory Comput 9: 3084–3095. 10.1021/ct400341p 26583988

[80] Miller BR III, McGee TD Jr., Swails JM, Homeyer N, Gohlke H, et al (2012) MMPBSA.py: An Efficient Program for End-State Free Energy Calculations. J Chem Theory Comput 8: 3314–3321. 10.1021/ct300418h 26605738

