# Supplementary figures and images for: Elucidation of Ligand-Dependent Modulation of Disorder-Order Transitions in the Oncoprotein MDM2
Source: PLoS Comput Biol. 2015 Jun 5;11(6):e1004282. doi: 10.1371/journal.pcbi.1004282 (PMC4457491; doi:10.1371/journal.pcbi.1004282)

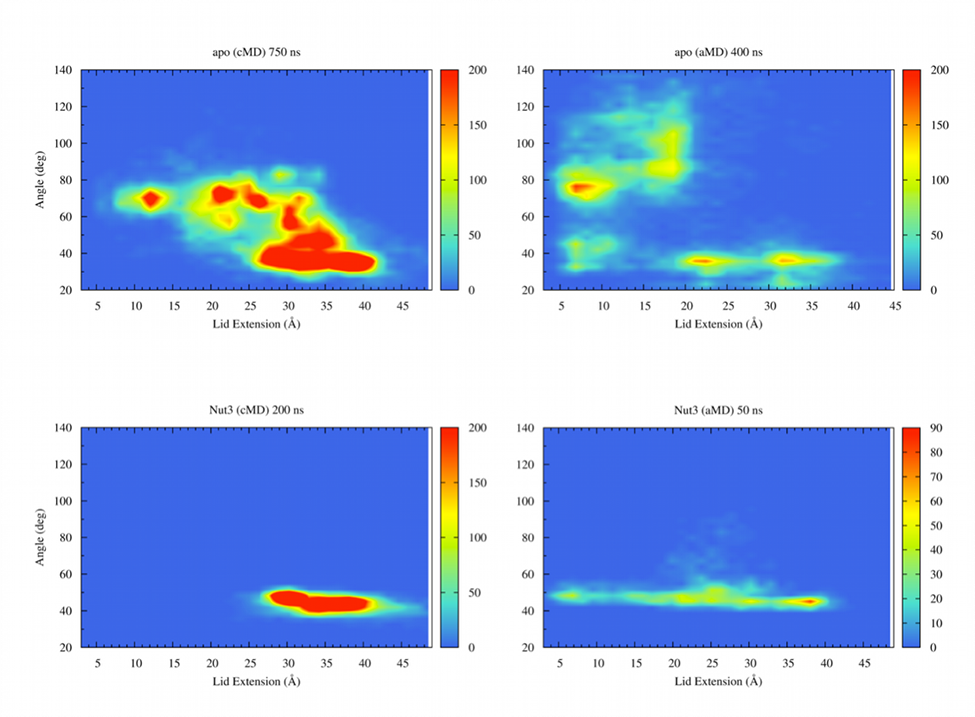

Supplement: S1 Fig — Top: Apo-MDM2, cMD (left) and aMD (right). Bottom. Nutlin3a-MDM2, cMD (left) and aMD (right). (TIFF) [file pcbi.1004282.s001.tiff]

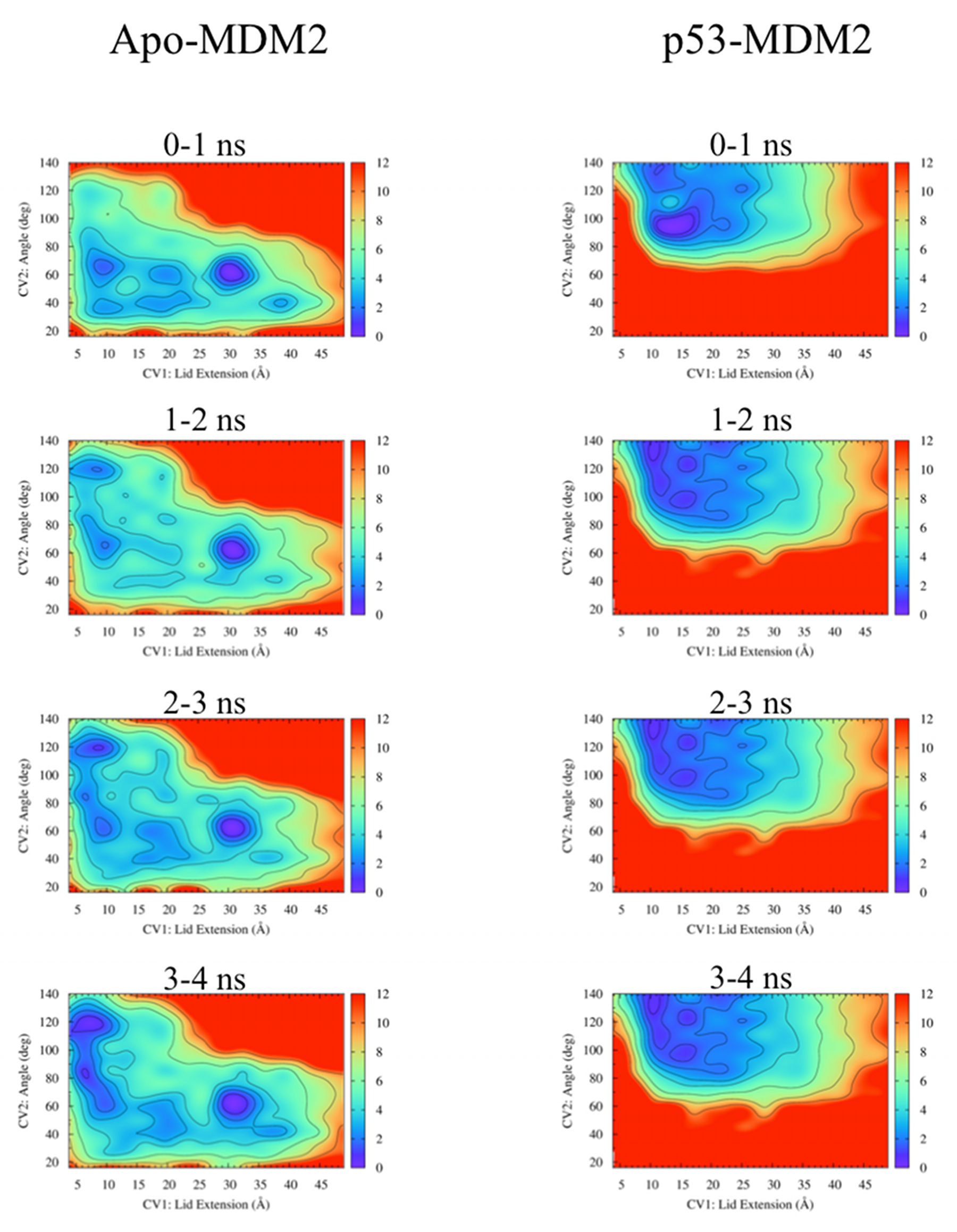

Supplement: S2 Fig — Energies are in kcal.mol-1. (Left) apo-MDM2 simulations. (Right) p53-MDM2 simulations. (TIFF) [file pcbi.1004282.s002.tiff]

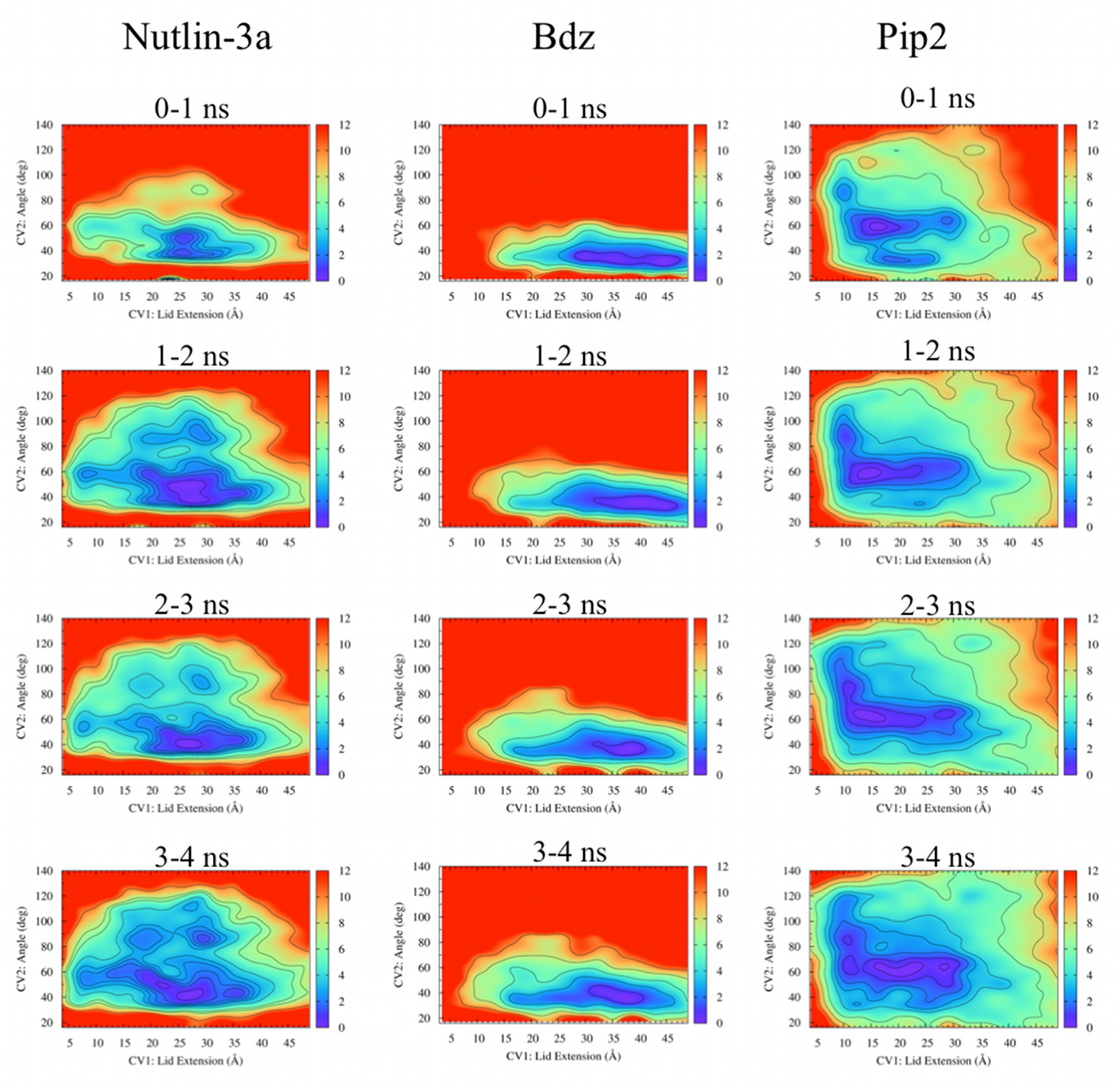

Supplement: S3 Fig — Energies are in kcal.mol-1. (Left) Nutlin3a-MDM2 simulations. (Middle) Bdz-MDM2 simulations. (Right) Pip2-MDM2 simulations. (TIFF) [file pcbi.1004282.s003.tiff]

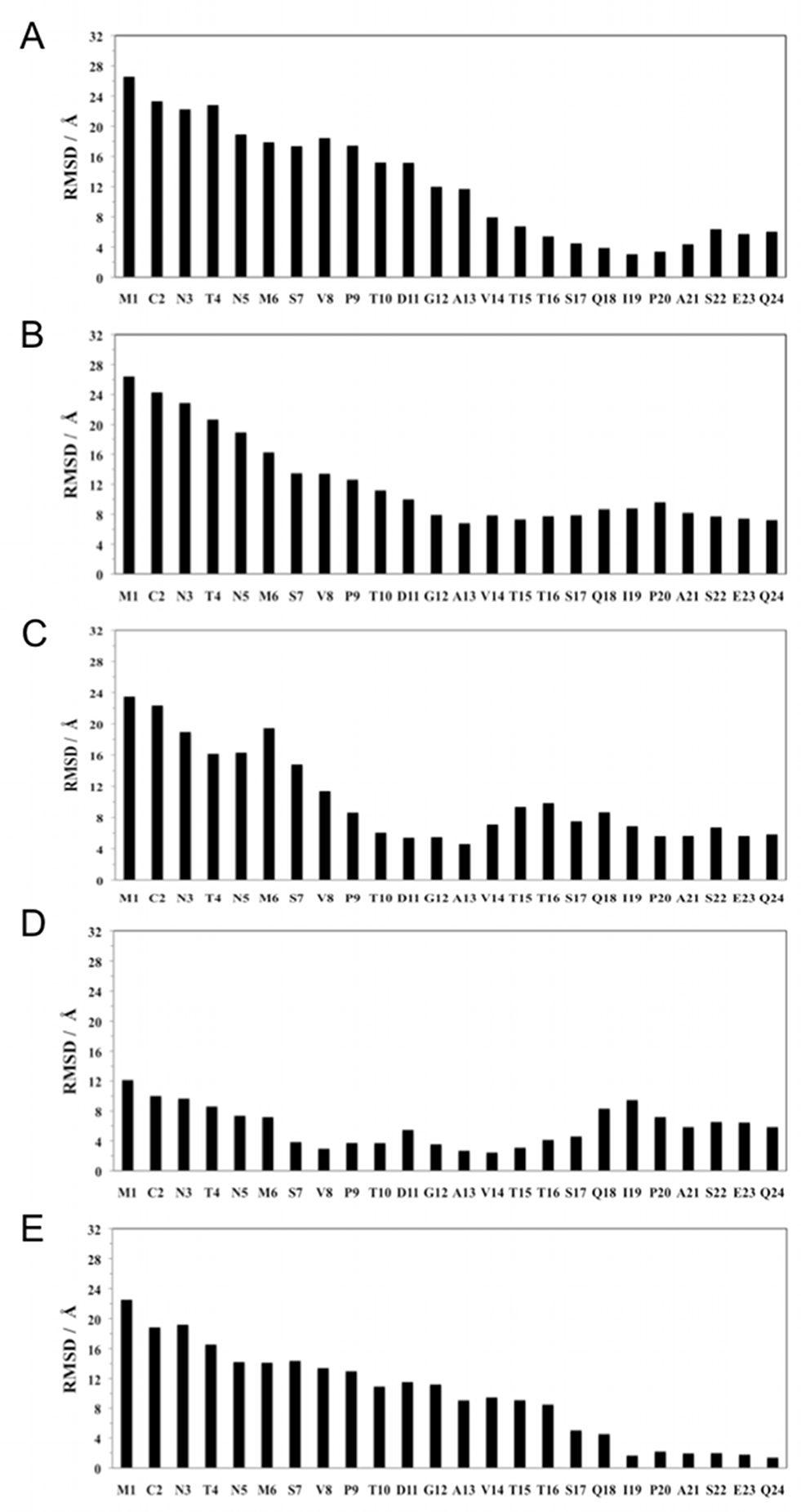

Supplement: S4 Fig — Per-lid residue average backbone RMSD in Å considering heavy atoms. A) apo, B) p53, C) Nutlin-3a, D) Bzd, E) Pip2. (TIFF) [file pcbi.1004282.s004.tiff]

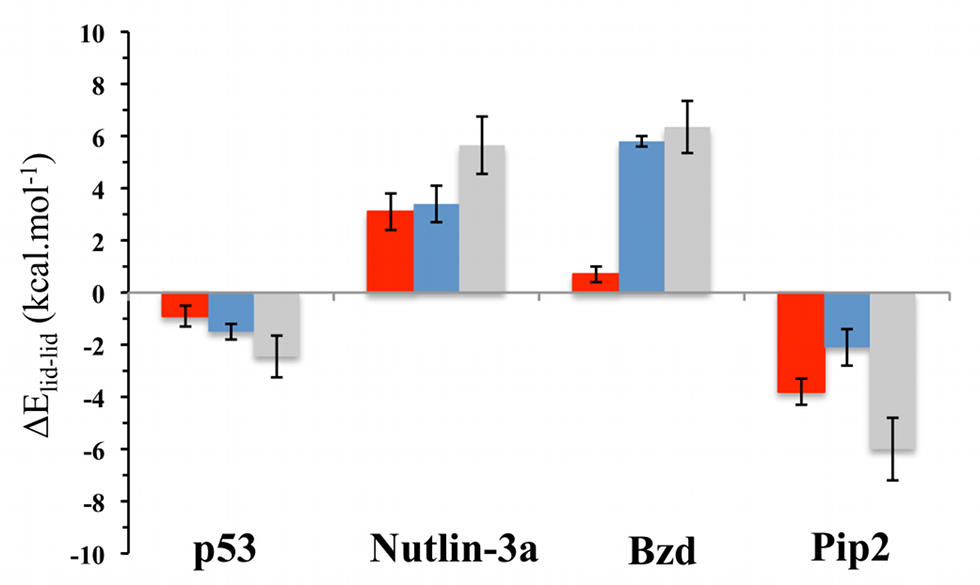

Supplement: S5 Fig — Lennard-Jones energies are depicted in red, Coulombic energies in blue and total interaction energies in gray. Energies are in kcal.mol-1. (TIFF) [file pcbi.1004282.s005.tiff]

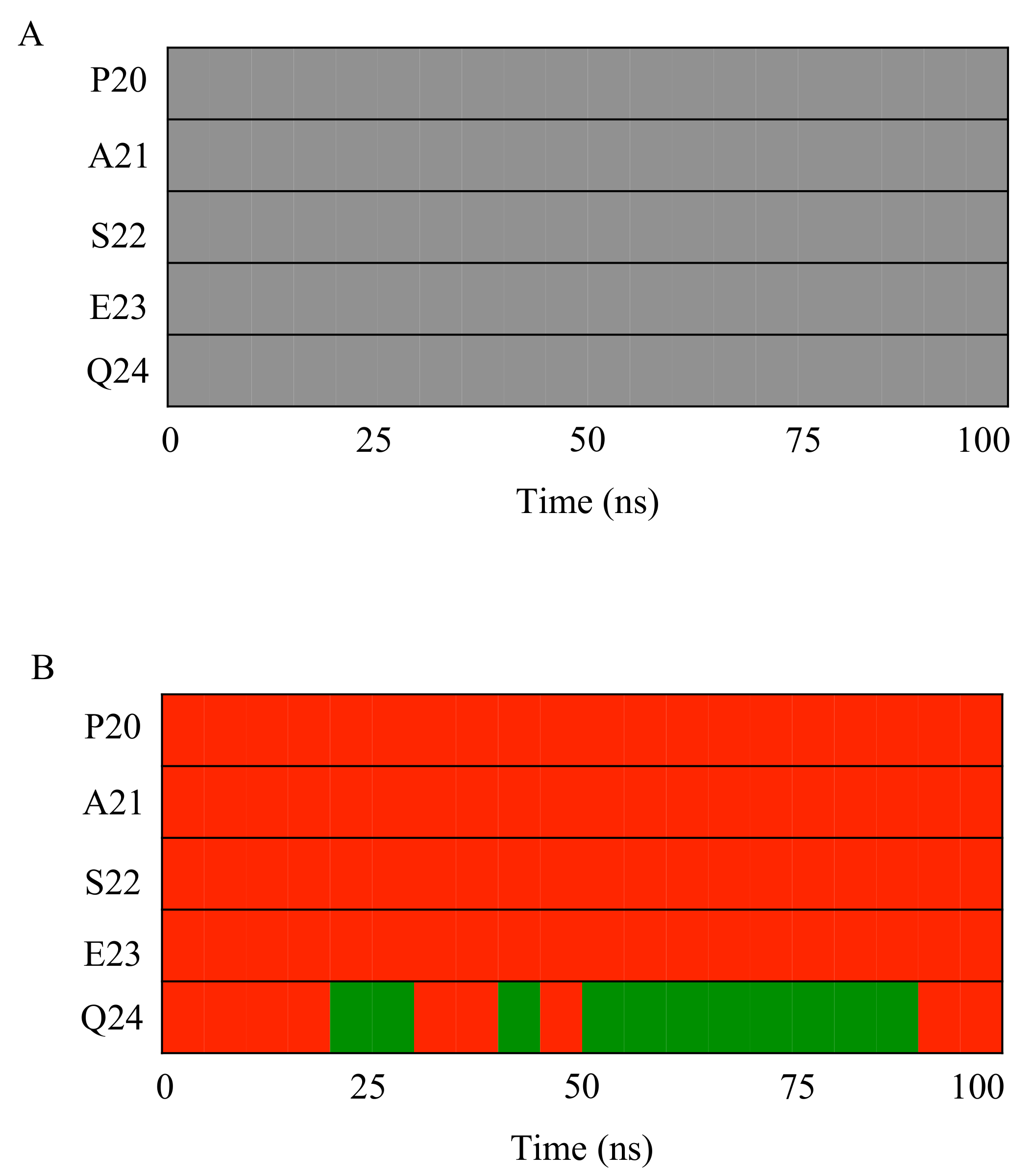

Supplement: S6 Fig — A) cMD simulations using as starting point a representative conformation from the minimum free energy well of the US of Nutlin3a-MDM2 complex (no α-helix displayed) (Residues 1–119). B) cMD simulations using as starting point a truncated form of the lid (Residues 17–25) starting from a previously formed α-helix between residues Pro20 and Gln24. Red: helix; Green: Turn; Grey: Coil. (TIFF) [file pcbi.1004282.s006.tiff]

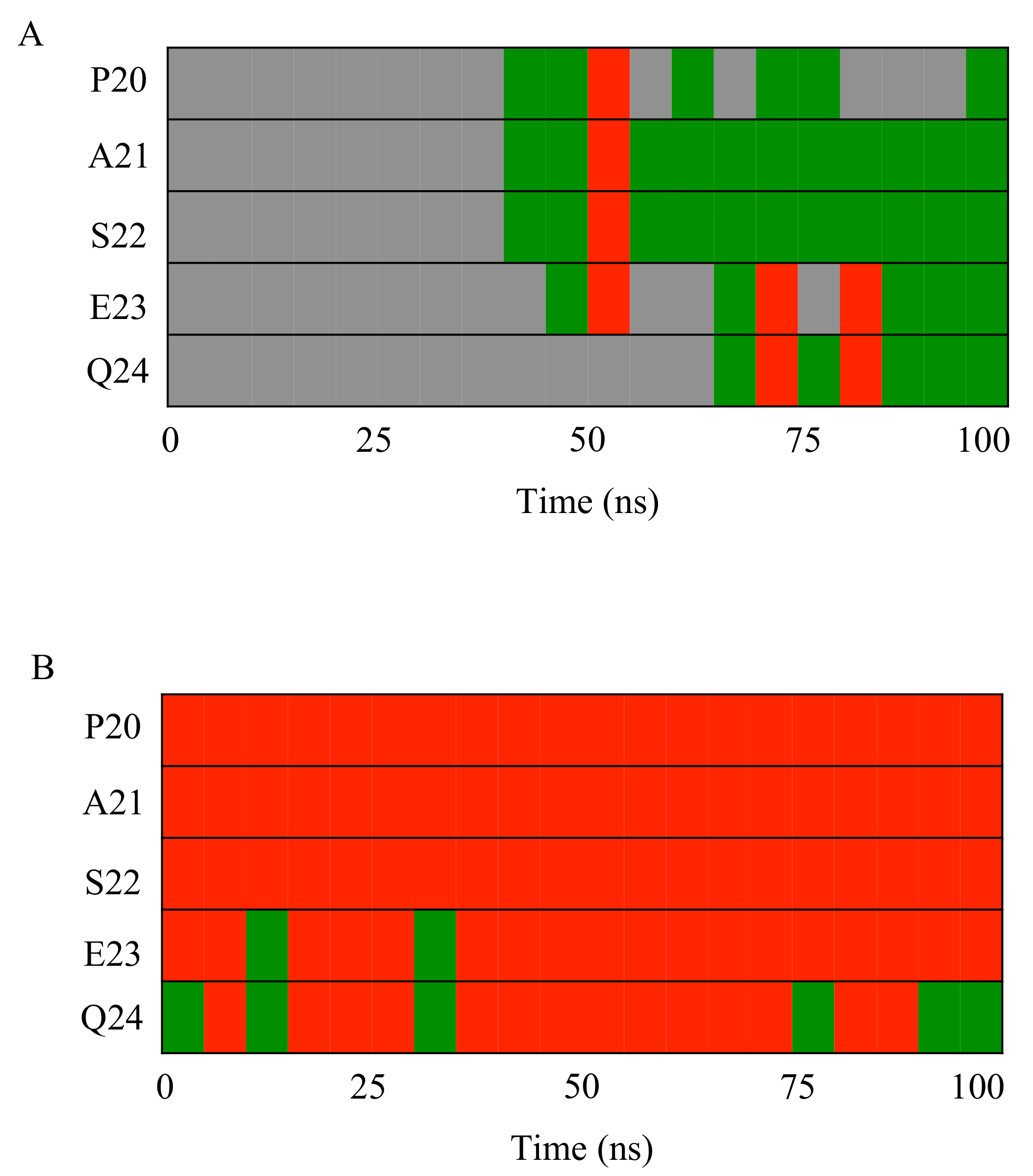

Supplement: S7 Fig — A) cMD simulations using as starting point a representative conformation from the minimum free energy well of the US of Bzd-MDM2 complex (no α-helix displayed) (Residues 1–119). B) cMD simulations using as starting point a truncated form of the lid (Residues 17–25) starting from a previously formed α-helix between residues Pro20 and Gln24. Red: helix; Green: Turn; Grey: Coil. (TIFF) [file pcbi.1004282.s007.tiff]
